# Telemedicine in the OECD: An umbrella review of clinical and cost-effectiveness, patient experience and implementation

**DOI:** 10.1371/journal.pone.0237585

**Published:** 2020-08-13

**Authors:** Nkiruka D. Eze, Céu Mateus, Tiago Cravo Oliveira Hashiguchi

**Affiliations:** 1 Division of Health Research, Health Economics at Lancaster, Lancaster University, Lancaster, United Kingdom; 2 Health Division Organisation for Economic Co-operation and Development, Directorate for Employment, Labour and Social Affairs, Paris, France; Queensland University of Technology, AUSTRALIA

## Abstract

**Introduction:**

Patients and policy makers alike have high expectations for the use of digital technologies as tools to improve health care service quality at a sustainable cost. Many countries within the Organisation for Economic Co-operation and Development (OECD) are investing in telemedicine initiatives, and a large and growing body of peer-reviewed studies on the topic has developed, as a consequence. Nonetheless, telemedicine is still not used at scale within the OECD. Seeking to provide a snapshot of the evidence on the use of telemedicine in the OECD, this umbrella review of systematic reviews summarizes findings on four areas of policy relevance: clinical and cost-effectiveness, patient experience, and implementation.

**Methods:**

This review followed a prior written, unregistered protocol. Four databases (PubMed/Medline, CRD, and Cochrane Library) were searched for systematic reviews or meta-analyses published between January 2014 and February 2019. Based on the inclusion criteria, 98 systematic reviews were selected for analysis. Due to substantial heterogeneity, a meta-analysis was not conducted. The quality of included reviews was assessed using the AMSTAR 2 tool.

**Results:**

Most reviews (n = 53) focused on effectiveness, followed by cost-effectiveness (n = 18), implementation (n = 17) and patient experience (n = 15). Eighty-three percent of clinical effectiveness reviews found telemedicine at least as effective as face-to-face care, and thirty-nine percent of cost-effectivenss reviews found telemedicine to be cost saving or cost-effective. Patients reported high acceptance of telemedicine and the most common barriers to implementation were usability and lack of reimbursement. However, the methodological quality of most reviews was low to critically low which limits generalizability and applicability of findings.

**Conclusion:**

This umbrella review finds that telemedicine interventions can improve glycemic control in diabetic patients; reduce mortality and hospitalization due to chronic heart failure; help patients manage pain and increase their physical activity; improve mental health, diet quality and nutrition; and reduce exacerbations associated with respiratory diseases like asthma. In certain disease and specialty areas, telemedicine may be a less effective way to deliver care. While there is evidence that telemedicine can be cost-effective, generalizability is hindered by poor quality and reporting standards. This umbrella review also finds that patients report high levels of acceptance and satisfaction with telemedicine interventions, but that important barriers to wider use remain.

## Introduction

In the last decade, several factors have contributed to a growth in the use of telemedicine. These factors include significant advances in information and communication technology, the prevalent use of high speed internet, and the increasing adoption of electronic health records. More recently, the SARS-CoV-2 pandemic has significantly accelerated telemedicine adoption and use, as patients and health care workers (HCWs) alike seek new ways to stay healthy with minimal physical contact. Telemedicine is used to encourage self-care through remote and chronic disease monitoring, to provide consultations to patients who are unable to attend in-person (face-to-face) appointments, and within hospitals and clinics to improve patient care. A key advantage of telemedicine is its ability to increase access to health care by offering patients the opportunity to receive care in their homes and communities. This umbrella review aims to provide a broad overview of how telemedicine is being used across OECD countries, focusing specifically on clinical and cost-effectiveness, patient experience and implementation.

## Objectives

To conduct a systematic review of the current literature on telemedicine and provide summary evidence of its effectiveness, cost-effective, patient experiences and implementation.

## Methods

### Eligibility criteria

We included only peer-reviewed published systematic reviews of primary studies that focused on one or more types of telemedicine (remote monitoring, real-time, and store-and-forward). Telemedicine is defined as the use of telecommunication systems to deliver health care at a distance [1]. Telemedicine is often used as a synonym for the boarder term, telehealth which encompasses both remote health care delivery and the delivery of education or training to patients and/or HCWs [2]. Other terms used to describe telemedicine include e-health, telecare, or m-health. Irrespective of the term used, we included all interventions that involved health care delivery through telecommunication systems that included either synchronous or asynchronous communication between a patient and their HCW. Therefore, reviews that focused on interventions such as automated text message reminders to improve medication adherence, or self-help websites with no direct interaction with a HCW were excluded.

We excluded non-English language reviews, reviews that focused on non-OECD countries, umbrella reviews, and reviews that did not clearly report on one or more of the predefined outcomes of this umbrella review: clinical effectiveness, cost-effectiveness, patient experience and implementation. For clinical effectiveness reviews, we further excluded reviews that did not report a pooled quantitative outcome (meta-analysis). For cost-effectiveness reviews, we included reviews of primary studies of economic evaluations or cost analyses.

### Search methods for the identification of reviews

Four online databases, PubMed/Medline, Centre for Reviews and Dissemination (CRD) and the Cochrane library were searched for systematic reviews or meta-analyses published between January 2014 and February 2019. Excluded citations and reasons for exclusion at full text eligibility stage are included in the supporting information (S1 File). Two review authors (NE) and (TCOH) designed the search strategy, data extraction template and conducted preliminary searches. One author (NE) performed the initial screening all titles and abstracts identified from the searches for inclusion based on the inclusion criteria and extracted initial data using a piloted data extraction form. Citations were coded as ‘included’, ‘excluded’ or ‘unclear’ as appropriate. Data was checked by (TCOH) and both authors agreed or disagreed on what reviews to include, and data to extract based on the protocol, and inclusion criteria. All disagreements were resolved by discussion. Due to heterogeneity of populations, interventions and outcomes in the included systematic reviews, no attempt was made to compare telemedicine interventions across reviews or across review populations.

### Assessment of methodological quality of included reviews

All three authors assessed the methodological quality of included reviews using the AMSTAR 2 tool [3]. The AMSTAR 2 is an update of the previous AMSTAR tool and it was chosen for its content validity, and its ability to assess systematic reviews of both randomised and non-randomised studies. Unlike the previous tool, the AMSTAR 2 is not designed to produce an overall score, rather reviews are graded according to methodological flaws in seven critical and nine non-critical domains.

### Data synthesis and unit of analysis

A meta-analysis was precluded due to heterogeneity of telemedicine interventions, intervention intensity, face-to-face comparators, study duration, HCW feedback methods and possible overlap of primary studies in the clinical effectiveness reviews. We presented results narratively, with tables and graphs for illustration where appropriate. The unit of analysis in this umbrella review is the systematic review and not the included primary studies, therefore only review level evidence was synthesised and included in findings.

## Results

**Fig 1:**
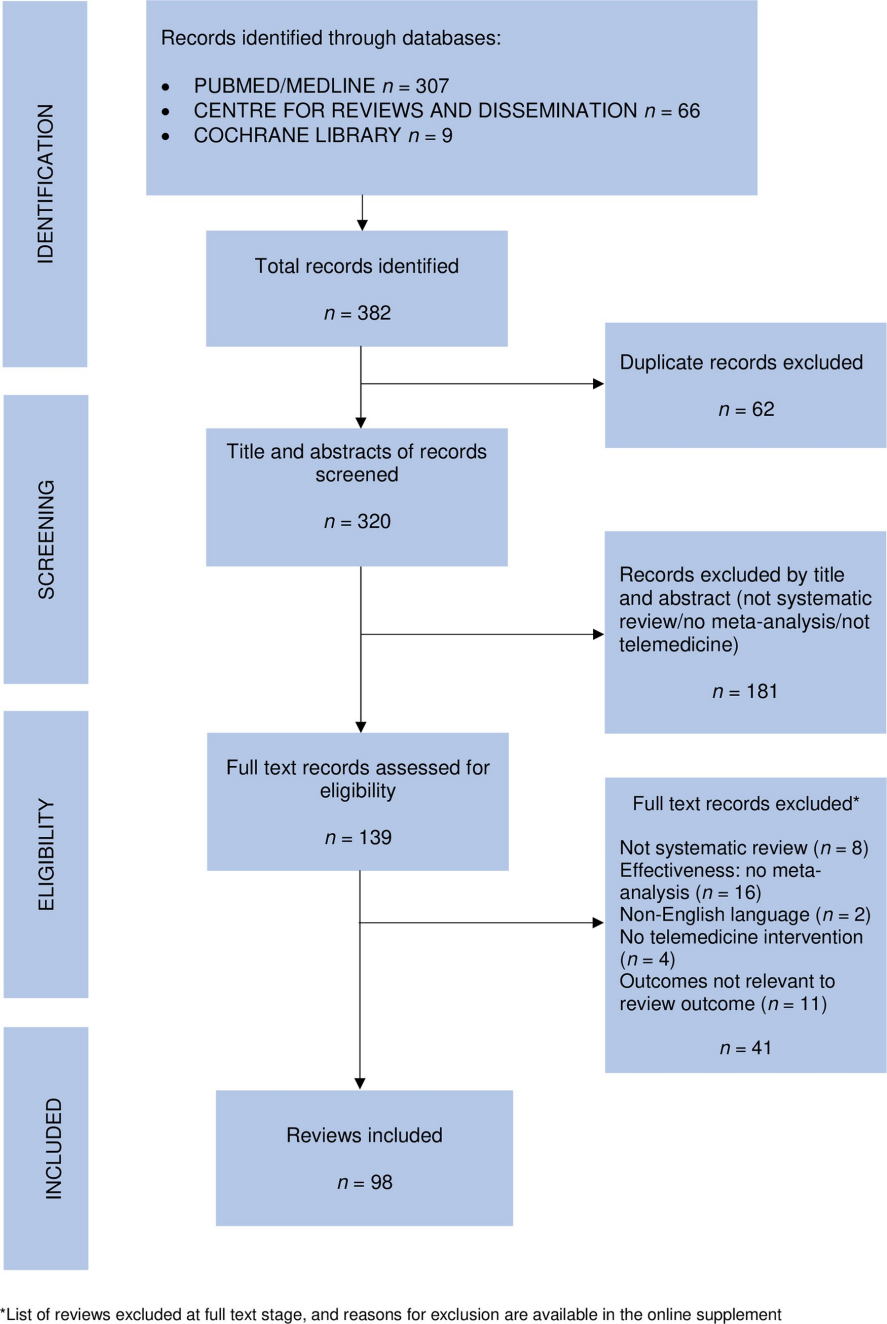
Prisma flow diagram.

The review selection process is summarized in a PRISMA flow diagram (Fig 1). The combined searches yielded 320 citations, and after exclusions 98 reviews were included in this umbrella review.

### Characteristics of included reviews

Characteristics of the 98 included reviews are summarized in the supporting information S2 File. All reviews were categorized according to the predefined outcomes of this review Table 1. Fifty-three reviews focused on clinical effectiveness, 18 on cost-effectiveness, 15 addressed patient experiences and 17 were on implementation of telemedicine. Five reviews addressed more than one outcome area and are marked as double listings.

**Table 1:** Classification of telemedicine reviews according to review outcome.

Review Outcome	Review [References]	*n*
**Effectiveness**	[4–53] [54]* [55]*	53
**Cost-effectiveness**	[51,55–69] [70]*[71]*[54]*[55]*	18
**Patient experience**	[72–84][85]*[71]*	15
**Implementation**	[86–100][85]*[70]*	17

*Double listings

#### Origin of primary studies included in reviews

**Fig 2:**
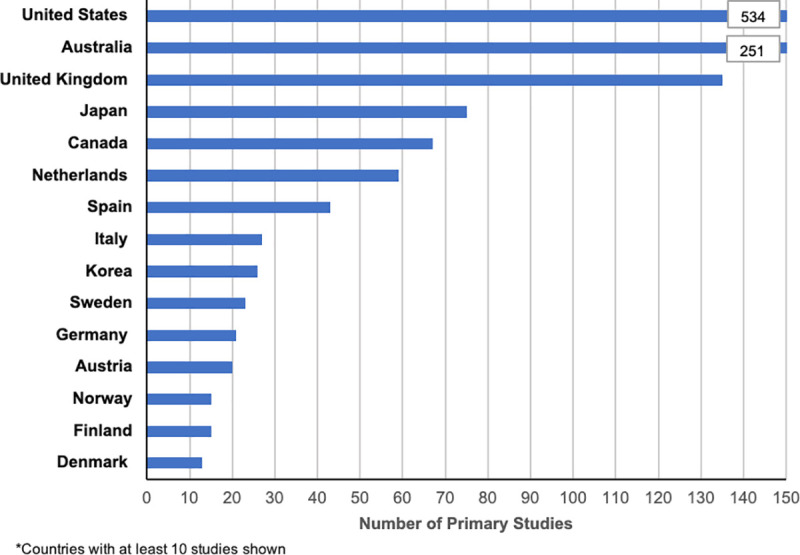
Origin of primary studies included in reviews.

Sixty-four reviews (65%) of included details of the origin of primary studies (Fig 2). The US had the highest proportion of primary studies 37% (534/1,456) followed by Australia 17% (251/1,456), UK 10% (135/1,456) and Japan 5% (75/1,456). The full list of countries and the number of studies from each country is included in the supporting information S2 File.

#### Description of telemedicine interventions

Telemedicine interventions included in this review are categorized as remote monitoring, interactive real-time telemedicine and store-and-forward systems. Remote monitoring involves asynchronous communication with monitoring devices, such as glucometers, blood pressure monitors, and mobile apps, which have the ability to transmit patient results remotely to HCW. Store-and-forward systems allow for the secure electronic transmission of patient data, and often take place between HCWs to aid diagnoses [4]. Real-time interventions use synchronous communication methods e.g. video and telephone consultations. These categories may be compared with usual care, an alternative telemedicine intervention, in addition to usual care, or no intervention. Table 2 includes the speciality area and type of telemedicine intervention used across the fifty three clinical-effectiveness reviews. The most frequently reported intervention used was device or app based remote monitoring in 75% (40/53) of reviews, while store-and-forward was the least frequent intervention reported in 4% (2/53) of reviews (Table 2). Three reviews included less conventional interventions such as virtual reality gaming [5] and social media [6,7]. A number of reviews included an element of patient education or training, however the primary aim of these interventions was health care delivery. All reviews except one addressed one or more specialty or disease areas. The sole review that didn’t focus on a speciality described the effect of telemedicine interventions delivered by allied health professionals and nurses in rural and remote areas [8].

**Table 2:** Effectiveness reviews by speciality area and type of telemedicine intervention.

First Author (Year)	Speciality Area	Remote Monitoring		Interactive Real-time		Store-and-forward
		Device or App based	Web-based*	Telephone Consults	Video Consults	
Adamse (2018)	Rehabilitation	✓	✓			
Agostini (2015)	Rehabilitation	✓	✓	✓		
Cottrell (2017)	Rehabilitation			✓	✓	
Cruz (2014)	Respiratory Medicine	✓				
Dario (2017)	General Medicine	✓	✓	✓		
Deady (2017)	Mental Health	✓	✓	✓		
Direito (2017)	General Medicine	✓				
Feltner (2014)	Cardiology	✓		✓	✓	
Flodgren (2015)	Cardiology Endocrinology	✓	✓	✓	✓	✓
Hakala (2017)	Rehabilitation	✓	✓	✓		
Huang (2015)	Cardiology	✓		✓		
Huang (2014)	Gastroenterology	✓				
Huang (2015)	Endocrinology	✓	✓	✓		
Hui (2017)	Respiratory Medicine	✓				
Hutchesson (2015)	Bariatric Medicine	✓	✓			
Jeon (2015)	Not specified	✓				
Joiner (2017)	Endocrinology	✓	✓	✓	✓	
Kelly (2016)	General Medicine	✓		✓	✓	
Kepplinger (2016)	Neurology				✓	
Klersy (2016)	Cardiology	✓				
Kotb (2015)	Cardiology	✓		✓	✓	
Lee (2016)	Paediatrics	✓				
Lee (2018)	Neurology			✓		
Linde (2015)	Mental Health		✓		✓	
Liu (2017)	Endocrinology Respiratory Medicine	✓				
Lundell (2015)	Respiratory Medicine	✓	✓	✓		
Marx (2018)	Geriatric Medicine	✓		✓		
McLean (2016)	Respiratory Medicine	✓				
Merriel (2014)	Cardiology	✓	✓	✓		
Ming (2016)	Obstetrics Endocrinology	✓				
Nair (2018)	Obstetrics Mental Health	✓	✓	✓		
Oosterveen (2017)	Mental Health	✓	✓	✓		
Raman (2017)	Obstetrics Endocrinology	✓				
Rasekaba (2015)	Obstetrics Endocrinology	✓	✓	✓	✓	
Rawstorn (2016)	Cardiology	✓	✓	✓		
Seiler (2017)	Oncology		✓			
Seyffert (2016)	Mental Health	✓	✓			
Sherifali (2017)	Obstetrics	✓	✓			
Speyer (2018)	Not specified		✓	✓	✓	
Stratton (2017)	Mental Health	✓				
Su (2016)	Endocrinology	✓	✓	✓	✓	
Tchero (2017)	Endocrinology				✓	✓
Thabrew (2018)	Mental Health		✓			
Thomas (2014)	Ophthalmology				✓	
Toma (2014)	Endocrinology		✓	✓	✓	
van Beugen (2014)	Mental Health	✓	✓	✓	✓	
van Egmond (2018)	Rehabilitation	✓	✓	✓		
Van Spall (2017)	Cardiology	✓		✓		
Vigerland (2016)	Paediatrics Mental Health		✓	✓		
Widmer (2015)	Cardiology	✓	✓	✓		
Wootton (2016)	Mental Health	✓	✓	✓	✓	
Zhai (2014)	Endocrinology	✓		✓		
Zhao (2015)	Respiratory Medicine		✓	✓		

*includes emails and online chat

#### Methodological quality of included systematic reviews

Overall, 8% (8/98) of reviews were graded as high quality, while the remaining 92% (90/98) were of low to critically low quality, S3 File. The most common critical weakness across reviews was the failure to include the full citation list of excluded studies with reasons for exclusion, followed by an explicit statement that the review was based on a prior protocol. Most reviews however performed adequate searches, used satisfactory techniques to assess methodological quality, and appropriate methods for meta-analysis where applicable.

In the non-critical domains, most reviews adequately followed and reported PICO elements, performed comprehensive literature searches, and reduced bias by selecting studies and extracting data in duplicate. The most common non-critical flaw across reviews was the failure to report sources of funding for included primary studies.

### Clinical effectiveness

#### Endocrinology

Eleven reviews evaluated the effectiveness of telemedicine interventions for diabetes management and all reviews concluded that telemedicine was superior or comparable to face-to-face interventions. Reviews compared telemedicine interventions to usual face-to-face care or no intervention and focused mainly on remote self-monitoring. One review focused on healing time for diabetic foot ulcers, using real-time video consultation and the second on the prevention or delay of Type 2 diabetes for at risk populations through remote monitoring weight loss interventions. [9,10]. The first review concluded that the telemedicine group had similar ulcer healing times as the face-to-face care group, while the second review found promising evidence of the efficacy of telemedicine with remote behavioral support for weight loss to prevent or delay Type 2 diabetes. Three out of the remaining ten reviews assessed the effectiveness of remote self-monitoring interventions on gestational diabetes. Two out of the three views were graded as high quality [11,12]. All three reviews found that telemedicine had similar clinical outcomes to face-to-face care for this population including glycemic control, cesarean delivery rates, pre-eclampsia, induction of labor and neonatal hypoglycemia [11–13]. The remaining seven reviews focused on glycemic control. One high-quality review found that remote monitoring can improve the control of blood glucose in diabetic patients [1], while the other six reviews concluded that remote monitoring was more effective than face-to-face care [14–19].

#### Cardiovascular disease

Eleven reviews assessed the effectiveness of telemedicine in cardiovascular disease management compared to usual care. In nine reviews, primary outcomes of interest across reviews included mortality, hospital readmissions, emergency room visits and risk factors measured by body mass index (BMI), systolic and diastolic blood pressure and low-density lipoprotein (LDL) cholesterol. Two reviews found that remote monitoring, and structured telephone support was superior to usual care in reducing the odds of mortality and hospitalization related to heart failure, improve survival rates and reduce cardiovascular disease risk factors such as weight, BMI and blood pressure [20,21]. Four reviews, including two high-quality reviews [1, 22] found no difference between remote monitoring with telephone support and face-to-face care to reduce planned hospital visits and to improve modifiable cardiovascular risk factors through cardiac rehabilitation [22–24]. One review produced mixed results, and found that telemedicine delivered through structured telephone support reduced heart failure specific readmissions but not all cause mortality readmissions [25]. Of the remaining two reviews, one concluded that telephone or remote monitoring interventions compared to nurse home visits had no significant improvement on readmission or mortality in heart failure patients [26], while the other found insufficient evidence that remote monitoring and counselling reduced overall cardiovascular disease risk [27].

The remaining two reviews assessed the effectiveness of telemedicine in stroke management. The first review investigated the safety and efficacy of treatment delivered through telestroke networks (real-time telemedicine) for patients with acute ischemic stroke compared to face-to-face care and concluded that telemedicine was safe and effective with no difference in mortality or functional independence between intervention and comparison groups [28]. The second review concluded that telephone consultations used in the management of oral anti-coagulation may lower the risk of major thromboembolic events, but not other clinically relevant outcomes [29].

#### Rehabilitation

Seven reviews assessed the effectiveness of telemedicine in rehabilitation (tele-rehabilitation) compared to usual care. The results were varied. For musculoskeletal conditions, video and telephone consultations were effective in the improvement of physical function compared to conventional care [30]. In surgical populations, wireless monitored exercise, web-based support and telephone consultations were at least as clinically effective as face-to-face care, with greater improvements in quality of life compared to face-to-face care [31]. For patients with chronic obstructive pulmonary disease (COPD), limited evidence suggested that remote monitoring with and telephone support could lead to improvements in physical activity [32]. For pain and disability, exercise monitoring, web-based programs and telephone consultations had no significant effect compared with usual care, but interventions that combined usual care and telemedicine were more effective than usual care alone [33]. In motor function, remote monitoring of exercise and training was effective for cardiac and orthopedic (total knee arthroscopy) patients, but the evidence was inconclusive for neurological patients [34]. For chronic pain, remote monitoring of excercise compared to no intervention, was effective in reducing pain and, furthermore, no difference was found in effectiveness between telemedicine compared to usual care for increased physical activity, or activities of daily living [35]. In cancer patients, therapist and self-guided web-based programs, and remote monitoring were effective for managing cancer-related fatigue [36].

#### Mental health

Ten reviews assessed the effectiveness of telemedicine interventions in mental health and behavioral conditions compared to usual care or no intervention. Nine studies concluded that telemedicine was at least as effective as face-to-face interventions, while one study was inconclusive. Eight studies had depression and/or anxiety as a primary outcome, while the remaining three focused on symptom reduction for obsessive compulsive disorder (OCD), insomnia, and reduction of alcohol consumption. From the eight depression studies, one focused on employees, one on maternal depression and two on children. The review that focused on employees observed a small positive effect for web-based congnitive behavioural therapy (CBT) with remote monitoring in reducing stress and anxiety in employees, but concluded that interventions targeted to individual employees were more effective than universal interventions [37]. For maternal depression, remote monitoring, and telephone consultations were effective in improving maternal depression symptoms [38]. For depression in children and adolescents, one high quality review found that the effect of multi-modal web-based CBT with support was inconclusive [39], while another review concluded that web-based CBT with structured telephone calls was effective in the treatment of psychiatric and somatic conditions [40]. Similarly, three other reviews found web-based CBT with remote monitoring, and remote therapist support was as effective as usual care in reducing symptoms of depression [41–43]. For adults with insomnia, web-based CBT with remote monitoring and real-time support was an effective therapy for improving sleep [44]. For OCD and behavioral interventions to reduce alcohol consumption in young adults, the effect of telephone counselling and remote monitoring was comparable to conventional face-to-face interventions [45,46].

#### Physical activity and diet

Four reviews assessed the effect of telemedicine on physical activity and nutrient intake. All four reviews found that telemedicine interventions were at least as effective as usual care. Targeted remote monitoring interventions were more effective than face-to-face care to increase physical activity, and universal interventions were comparable to face-to-face interventions in reducing sedentary behavior [47,48]. Remote monitoring, video and telephone consultations could improve diet quality, as well as fruit, vegetable, and dietary sodium intake for people with chronic conditions and malnourished community dwelling older adults [49,50].

#### Weight loss

Four reviews investigated the effect of telemedicine interventions on weight loss. The first review found that remote monitoring alone achieved modest weight loss compared to no intervention, but achieved significantly greater weight loss when combined with behavioral features [51]. The second review found that app-based remote monitoring interventions in elementary school children had no significant effect for weight control, exercise, or sugar sweetened beverage intake [52]. The third review found a small positive effect of app-based remote monitoring for weight loss [19]. Finally, for weight loss during pregnancy and the postpartum period, remote monitoring interventions were only effective in postpartum women [53].

#### Respiratory disease

Four reviews investigated the effectiveness of telemedicine for asthma control and COPD. Two reviews found telemedicine was at least as effective as face-to-face care, and two were uncertain about the effectiveness of telemedicine. The first review concluded that mobile app-based remote monitoring interventions which facilitated professional support improved asthma control and exacerbation rates [54]. The second review found no difference in asthma symptom scores between the intervention groups, (remote monitoring and telephone consults) and face-to-face care groups [55]. The third review concluded, using low quality evidence, that remote monitoring interventions had small beneficial effects on asthma control [56], while the fourth found limited evidence of the effect of home based remote monitoring to reduce health care utilization, quality of life and respiratory exacerbations in COPD patients [57].

#### Other specialties

One review assessed the effectiveness of telemedicine in the management of inflammatory bowel disease (IBD). The authors concluded that remote monitoring interventions reduced clinic visit utilization compared to face-to-face care, and were as effective as face-to-face care in other outcome areas including relapse rates, hospital admission rates and quality of life [58].

Remote monitoring, video and telephone consultations delivered by allied health professionals to patients in rural areas were at least as effective as face-to-face interventions [59]. For glaucoma, tele-glaucoma via video consultations was more effective than face-to-face examinations in detecting glaucoma besides reducing waiting times, offering services to rural areas, and reducing travel [60].

### Cost-effectiveness

Eighteen reviews assessed the costs or cost-effectiveness of telemedicine interventions. Seven reviews found that telemedicine was cost-effective or cost saving, Table 3.

**Table 3:** Summary charactheristics of cost-effectiveness reviews.

Author (Yr.) Country	a. Speciality	a. Intervention	Conclusions
		b. Perspective	
		c. Time horizon (Range)	
	b. Included Studies		
Akiyama (2016) Japan	a. Geriatrics	a. RM, VC urban and rural homes	TM programs in Japan appear to have a favourable level of economic efficiency.
	b. 11(1 CMA, 2 CBA, 4 WTPE, 4 BE)	b. Provider, Societal	
		c. 30 days to 11 yrs.	
De la Torre-Diez (2015) Denmark	a. Dermatology, Cardiology, Endocrinology, Mental Health, Ophthalmology	a. RM, VC rural areas, inmates	Variation across studies made it unrealistic to draw an overall conclusion about cost-effectiveness of TM
		b. Provider	
		c. n/s	
	b. 35(CUA, CEA)		
Estai (2018) Australia, UK	a. Dentistry	a. VC screening for oral diseases	VC can be cost-saving compared to a F2F care, however there is inconclusive evidence to support evidence-based policy decisions
		b. Provider, Patient	
	b. 2 CMAs		
		c. n/s	
Fuertes-Guiro (2017) UK, Austria, Italy	a. Dermatology	a. Teledermatology (TD): VC, S&F	TD lasted 7.5 mins more on average than F2F consultation, increasing costs but there is no unamity due to incomplete cost reporting
	b. 8 RCTs	b. Provider, Patient	
		c. n/s	
Grustam (2014) US, Australia, Italy, China, Taiwan	a. Cardiology	a. RM, TC to manage CHF patients	60% of high-quality studies found TM cost-effective, but cost-effectiveness is uncertain due to poor and scarce economic evidence
	b. 32(21 RCTs, 2 QRCTs,9 NRs)	b. Provider	
		c. 78% < 1 yr.	
Hammed (2014) UK, USA, Germany, Brazil, New Zealand, Taiwan	a. Heart Failure	a. RM, TC for heart failure patients	There is currently no evidence that increased patient adherence, supported by TM, has led to a reduction in treatment costs for HF patients.
		b. n/s	
	b. 9 (2 RCTs, 1 QRCT, 2 CEA, 3NRs)		
		c. n/s	
Irribarren (2017) US, UK, Africa, Europe, China, Thailand, Australia	a. Cardiology, Diabetes	a. RM with mobile devices	Findings support the cost-effectiveness of Mhealth interventions, but this result must be considered cautiously, and evaluated on a case-by case basis
		b. Provider, Societal	
	b. 39 (25 CEA, 12 CUA, 1 CMA, 1 CBA)		
		c. 30 days to Lifetime	
Jackson (2017) UK, Denmark, Ireland	a. Gastroenterology	a. RM, VC	Studies described cost savings, but did not account for indirect intervention costs. Further work is required to appreciate whether or not TM is cost-effective
		b. n/s	
	b. 2 (1 RCT, 1 NRs)	c. n/s	
Liddy (2016) Spain, Netherlands, USA	a. Dermatology, Paediatrics, Multispeciality	a. Teledermatology (TD): VC	TD has the potential for large cost savings, but gaps remain due to variation in reporting which limits comparability.
		b. Provider, Societal	
	b. 7(2 CA, 3 CMA, 2 CEA)		
		c. n/s	
Lopez-Villegas (2015) UK, US, Canada, Italy, Austria, France	a. Cardiology	a. RM, pacemaker monitoring	Pacemaker RM detects cardiovascular events earlier reduces hospitalizations, the number of hospital visits, and associated follow-up costs.
	b. 7 (4 CEA, 1 CBA, 1 CUA, 1 CMA)	b. Provider, Societal	
		c. 10 months to 7 yrs	
McDougall (2017) Australia, UK, Canada, Italy, US	a. Internal Medicine	a. Telerheumatology: VC, TC	All included studies found within a limited body of evidence that TM was cost-effective for the management of rheumatic disease
	b. (6 CA)	b. n/s	
		c. n/s	
Michaud (2018)	a. Diabetes, Heart Failure, Cancer, COPD	a. Home-based monitoring: VC, RM	Although all studies found that TM reduced costs, a comprehensive analysis of costs and their determinants is still required for wider adoption and reimbursement of TM
		b. Provider	
	b. 12 (6 RCT, 2 QRCTs, 4NRs)		
		c. n/s	
Musiat (2014) n/s	a. Mental Health	a. cCBT: RM, VC	Overall results suggest that cCBT is a cost-effective alternative to usual care with similar or superior results at lower direct costs.
		b. Societal, Provider	
	b. 13 (5 CUA, 8 CEA)		
		c. n/s	
Sanyal (2018) Australia, Canada, Netherlands, Spain, US, UK	a. Cardiology, Diabetes, Mental Health	a. RM, TC, VC, for older adults	There is a lack of convincing evidence to conclude whether TM to deliver health care to older adults will demonstrate value at any acceptable level of investment.
		b. Provider	
	b. 11 (CEA, CUA)		
		c. 4 months to lifetime	
Snowswell (2016) US, UK, New Zealand, Netherlands, Spain	a. Dermatology	a. Teledermatology: S&F	Current evidence is sparse, but suggests that TD can be cost-effective
		b. Provider, Societal	
	b. 11 (4 CMA, 4 CEA, 2 CUA, 1 CA)		
		c. n/s	
Thomas (2014) Germany, Canada, Finland, Indonesia, Taiwan, US, Spain	a. Opthalomologyb. 9 (3 CEA, 6 NRs)	a. Teleglaucoma (TG): remote areas	TG considerably reduces patient access time, and physician consultation time, thereby saves costs to patients and the healthcare system as a whole.
		b. n/s	
		c. n/s	
Udsen (2014) Canada, US, Italy, Spain, Denmark	a. Respiratory Medicine	a. RM: COPD patients	Limited evidence showed a potential for cost savings with lower average cost per patient with TM plus usual care vs. usual care alone.
	b. 6 (1 CUA, 3 CCA, 2 CMA)	b. Provider	
		c. 3–12 months	
Zhai (2014) n/s	a. Endocrinology	a. RM: Type 2 diabetes patients	The two studies reported wide ranging costs, confounding attempts to draw meaningful conclusions about cost-effectiveness
		b. n/s	
	b. 2 (1 RCT, 1 CEA)		
		c. n/s	

TM = Telemedicine, CUA = Cost Utility Analysis, CCA = Cost Consequence Analysis, CMA = Cost Minimization Analysis, CEA = Cost Effectiveness Analysis, RCT = Randomized Controlled Trial, S&F = Store-and-forward, RM = Remote Monitoring, TC = Telephone Consultation CA = Cost Analysis, NHS = National Health Service, WTPE—Willingness to Pay Estimation, BE = Benefit Estimation, RM = Remote Monitoring, VC = Video Consultation, F2F = Face to face, QRCTs = Quasi-randomised Controlled Trials, NRS = Non-randomized Studies, CHF = Chronic Heart Failure, N/S = Not Stated, cCBT = computerized cognitive behavioural therapy

For the management of rheumatoid arthritis video and telephone consultations were found to be cost-effective compared to usual face-to-face care [61]. In mental health, a review assessed computerized cognitive behavioral therapy (cCBT) delivered through remote monitoring and video consultations from a provider or societal perspective. They found that cCBT was cost-effective compared to face-to-face care mainly because the time spent on cCBT interventions were up to six times lower compared to face-to-face therapy, thereby reducing the workload of mental health practitioners [62]. For tele-glaucoma, the time spent by the physician compared to time spent in face-to-face examination was shorter, and as a result tele-glaucoma was cost-effective, offering a potential saving of $175.9 USD per patient compared to conventional face-to-face diagnosis [60]. In Japan, remote monitoring and video consultations were found to be generally cost-effective for teledermatology and teleradiology from a provider or societal perspective. However tele-homecare produced mixed results due to lower willingness to pay and higher system costs in areas with public subsidies, which led to overutilization [63]. In one high-quality review, 74% of included studies found that remote monitoring through mobile devices to support data collection was cost-effective, economically beneficial, or cost saving at base case from a provider or societal perspective [64]. Compared to hospital monitoring, pacemaker remote monitoring interventions were cost-effective from a provider or societal perspective with no significant difference between groups in quality of life or adverse events [65]. Store-and-forward teledermatology, as an adjunct to conventional face-to-face care may be cost-effective from a provider or societal perspective especially when used as a triage tool to reduce waiting times as well as the costs and time associated with travel to face-to-face appointment requirements [66].

Five reviews concluded that although telemedicine interventions could be cost-effective, or cost saving, heterogeneity, poor quality and paucity of cost data limited the ability to arrive at a more robust conclusion. In one review of home-based monitoring, costs varied substantially depending on specific chronic conditions, and ranged from $1,352 USD for heart failure to $206,718 USD for congestive heart failure, COPD and diabetes as a whole. While cost data was limited, the available data indicated that remote monitoring and video consultations were cost saving from a provider perspective [67]. The second review concluded that remote monitoring and video consultations could potentially be cost-effective from a provider perspective, however insufficient high-quality evidence precluded a more certain conclusion [68]. The remaining three reviews found that cost-effectiveness of video consultations and remote monitoring was uncertain due to low quality and insufficient evidence. The first review included studies that performed analysis from a provider or patient perspective for tele-dentistry [69], while the other two reviews were from a provider perspective for chronic heart failure [70] and COPD [71].

Two reviews were unable to arrive at a conclusion regarding cost-effectiveness due to variation in outcomes within included primary studies. The first review included studies that performed analysis from a provider and societal perspective and concluded that heterogeneity in outcome reporting limited the ability to draw conclusions on the cost-effectiveness of teledermatology. [72] Included studies in the second review produced very different incremental cost-effectiveness ratio (ICER) values of $491 and $29,869 for remote monitoring in patients with Type 2 diabetes [18].

Four reviews concluded that based on the available evidence, telemedicine interventions were not cost-effective. The first review concluded that it was doubtful, based on current evidence, that the use of remote monitoring, video or telephone consultations to deliver health to older adults could demonstrate value at any acceptable level of investment from a providers perspective [73], while the second found using limited evidence that remote monitoring and video consultations may increase costs [74]. The third review included studies that performed analysis from a provider and patient perspective and concluded that teledermatology takes more time than face-to-face consultations presenting higher costs than face-to-face care, and thereby increasing the total cost of consultations [75]. Finally, the fourth review found no evidence that increased patient adherence supported by remote monitoring and telephone calls led to a reduction in treatment costs for heart failure patients [76].

### Patient experience

Fifteen reviews focused on patient experiences with telemedicine. Three main themes were identified: patient acceptability, patient satisfaction and patient barriers to the use of telemedicine.

#### Patient acceptability

Six reviews addressed patient acceptability of telemedicine. All reviews found that telemedicine interventions were acceptable to patients. In primary care settings, remote monitoring and real-time telemedicine interventions are generally acceptable, however the level of acceptability varied by population demographics such as gender, age and socio-economic status. Furthermore, these interventions were more acceptable to patients than to health care providers [77]. Mobile-app based remote monitoring interventions were acceptable to children and adolescents with mental health conditions [78]. For people with severe mental illness, web or mobile-phone based remote-monitoring was acceptable, with higher acceptability levels when participants were provided with remote online support [79]. For the secondary prevention of cardiovascular disease, app-based remote monitoring interventions were feasible and acceptable to cardiac patients with high levels of engagement, usage and adherence [80]. Finally, remote-monitoring, real-time telemedicine, and store-and-forward interventions were acceptable to COPD and dermatology patients [81,82].

#### Patient satisfaction

Six reviews addressed patient satisfaction and enablers to the use of telemedicine interventions. For patients with mental health conditions, text-messaging interventions improved treatment adherence and symptom surveillance, and increased patient satisfaction with management and health care services [83]. Mental health patients who had undergone computerized cognitive behavioural therapy (cCBT) reported high treatment satisfaction rates, however more research was advised to address attrition due to dissatisfaction [62]. COPD patients reported that they were satisfied with remote monitoring, and that they found interventions useful to help manage their condition [84]. Cancer patients and survivors had a positive experience with remote monitoring and telephone consultations. They reported that interventions were convenient, acceptable, fostered independence and remote reassurance, reduced burden and provided the safety net of a professional health care connection [85,86]. Telemedicine interventions incuding remote monitoring and real-time telemedicine improved social and emotional wellbeing of indigenous people receiving care in the community, improved patient outcomes and access to specialist services, and resulted in greater patient empowerment due to improved health literacy [87].

#### Barriers to patient uptake and use

Four reviews reported patient barriers to the use of telemedicine. The first review concluded that lack of training, poor patient tolerance with faulty systems, and technical problems all negatively affected uptake and sustained use of home-based remote monitoring [88]. The second review, was graded as high-quality and focused on the experiences of young people with chronic non-communicable diseases. The authors concluded that lack of collaboration between implementers and end users, as well as an inability to tailor and adapt technology to meet person-centered needs and preferences, were barriers to the wider use of mobile or app-based remote monitoring interventions [89]. For COPD patients, the third review found that remote monitoring devices with reported technological and usability problems had lower intervention compliance rates [84]. In heart failure patients, low patient motivation, lack of confidence and a preference for face-to-face interventions were all barriers to the use of remote monitoring interventions, resulting in poor uptake of interventions [90].

### Implementation

Seventeen reviews reported on the implementation of telemedicine. The main themes identified were barriers, enablers, risk factors, feasibility and sustainability.

#### Barriers to implementation

Three reviews reported on barriers to the implementation of telemedicine. Three reviews identified barriers to remote monitoring in the management of diabetes, including poorly designed interfaces, manual data input, transmission with delayed feedback, limitations on scalability, and technological illiteracy [91,92]. These factors contributed to recall bias or human error which impeded patient engagement, resulting in large drop-out rates [92]. In indigenous communities, barriers to telediabetes included the lack of technical skills and shortage of local staff [93]. Cost factors included start-up costs, ongoing costs, and costs related to loss of revenue. The absence of or inadequate legislation and policies, and liability cover might constitute a barrier to implementation at organization and health professional level. Furthermore, incompatibility with health systems, work practices or daily clinical work leading to disruptions to workflow and delivery of care were common barriers to implementation. Finally, lack of strategic planning and engagement of key stakeholders in development, selection and adoption of electronic health (eHealth) systems, were also problematic.

#### Enablers of successful implementation

Five reviews reported on enablers of telemedicine implementation. The first review concluded that interventions should be targeted, and systems designed to solve clinical or behavioral problems identified by patients as priorities. Furthermore, telemedicine technology should be automated, streamlined, mobile and low-cost to limit the burden of usage on patients, and maximize clinical utility [91]. The second review identified tailoring home-based remote monitoring interventions to patient characteristics and needs, relationship and communication between patient and health care professionals, technology usability and quality, and home health care organization, as enablers of telemedicine [94]. For indigenous populations, the involvement of indigenous health workers was essential in delivering telediabetes interventions to these communities because of their role in communication in local language and the potential to help clinicians understand the local community and facilitate integration [93]. In mental health, understanding treatment preferences, facilitating uptake and governing mechanisms were identified as enablers to implementation [95]. For diabetic patients, interventions incorporating elements of structured monitoring of blood glucose identified by the International Diabetes Foundation as essential for improving A1c (average blood glucose level over the past 3 months) were associated with comparatively significant improvement in clinical outcomes. These elements included patient and provider education, the use of pre-approved structured monitoring, goal setting, shared decision making and interactive communication [96].

#### Risks associated with using telemedicine

One review reported on patient risk factors associated with home-based remote monitoring and found that patient safety risks were mainly attributable to a lack of patient and or staff knowledge and understanding, changes in the nature of clinical work, technological issues, and patient dependency or anxiety [97]. Problems with telemedicine technology and devices, organizational issues and environmental factors could also contribute to patient safety risks but to a lesser extent. Risk concerns were mainly raised by health care professionals [97].

#### Sustainability of telemedicine initiatives

Four reviews addressed the sustainability of telemedicine. In rural and remote Australia, vision, ownership, adaptability, and economic efficiency aided sustainability while contributing to equity of access to health care [98]. For older adults, the success of remote monitoring interventions to monitor and improve their health greatly depended on the support and training they received [99]. Staff, technical and service factors also had an impact on the sustainability of remote monitoring interventions [90]. Staff factors associated with unsustainability included the absence of champions, dislike of new clinical routines or interactions, perception of no value or compromised clinical expertise. Technical factors that made telemedicine service likely unsustainable include unreliable or difficult technology and inadequate helpdesk support, while service factors included a lack of clarity on who was responsible for interpreting or actioning remote monitoring data. The fourth review found that the renumeration of participating clinicians was crucial to the sustainability of real-time and store-and-forward telemedicine interventions [100].

#### Feasibility of telemedicine interventions

Seven reviews reported on the feasibility of telemedicine interventions. Feasibility includes clinical, organizational or technical factors determining the practicality of implementing telemedicine services. Remote monitoring and real time telemedicine was feasible in patients with neurological diseases [101], in primary care settings [77]. Real-time and store-and-forward intervenitons were feasible in dentistry [100] and for remote monitoring was feasible in Japan [102]. A higher level evidence was required to support clinical application of teledermoscopy for diagnostic measurement in the treatment of precancerous and cancerous skin lesions [103]. Feasibility of telemedicine depended on partnerships between researchers, health care planners and policymakers to help align implementation research with policy development and funding [104]. The final review concluded that for older adults, usability and costs are key to feasibility [105].

## Discussion

This umbrella review identified a diverse and growing body of literature on telemedicine, and provides a broad summary of the use of telemedicine within the OECD. Eighty-three percent (44/53) of effectiveness reviews found that telemedicine interventions were at least as effective as face-to-face care. In disease areas such as diabetes management, all included reviews found that telemedicine interventions were effective. Two (4%) of reviews found that telemedicine was ineffective compared to face-to-face care, and seven (13%) reviews were uncertain about the effect of telemedicine. Although most reviews found positive effects for the use of telemedicine, these findings should be interepreted cautiously, given heterogenetiy across populations, interventions, settings and the overall low quality of incuded reviews.

A wide range of terms were used to describe telemedicine interventions such as telehealth, mhealth, and ehealth, confounded by terms specific to disciplines and specialities such as telenursing, teledermatology and telerehabilitation. This inconsistent use of terms may impede knowledge translation and dissemination. Categorizing interventions by type of telemedicine used (e.g. video consultations vs remote monitoring) may be useful to provide a more targeted summary. It may also be helpful when pooling results, to conduct sub-group analysis according to intervention type, and intensitiy.

The impact of interventions on utilization of health care was not examined. Although telemedicine may reduce the demand for face-to-face care, some evidence suggests that telemedicine, especially direct-to-consumer (DTC) telemedicine is often used as an adjunct to face-to-face care and may increase health care utilization and costs [106].

Seven out of the 18 (39%) included reviews on cost-effectiveness concluded that telemedicine interventions were cost-effective or cost saving compared to usual care. Five out of 18 reviews (28%) found that telemedicine might be cost-effective or cost saving, two out of 18 reviews (11%) were unable to arrive at a robust conclusion, and four out of 18 reviews (22%) found that telemedicine was not cost-effective compared to usual care.

Most of the reviews on cost-effectiveness reported shortcomings in the economic literature on telemedicine including a general paucity of economic studies, poor quality of available primary studies, poor outcome reporting, and lack of cost data. Some reviews included partial economic evaluations (e.g. cost anaylsis) which fails to provide the requisite detail for cost-effectiveness, therefore the evidence from these reviews were limited.

Six reviews included economic evaluations that performed analysis from a societal perspective. This perspective is considered high quality because it includes both direct and indirect costs that may be saved or incurred by patients and providers irrespective of who bears the costs or receives the benefits. Ideally, a societal perspective should be combined with a budget impact assessment, which will help improve the feasibility of implementing interventions [71]. Most reviews included analysis from a provider perspective, where only costs to the health care system were included and costs that may be incurred by patients and carers were excluded, such as for example, travel time, productivity or wage loss, accommodation costs or co-payments [66]. For the reviews that included cost analysis, some studies failed to take into account the cost of treatments or devices already in place, and implementation costs [61,74]. Five reviews included time horizons from economic evaluations. Time horizons ranged from thirty days to lifetime, see Table 3. Longer time horizons are often useful, especially when evaluating chronic conditions where costs and outcomes are likely to incur later. However, telemedicine is a rapidly evolving field, and longer time horizons may become meaningless as the telemedical technology becomes obsolete. Therefore, infrastructure costs such as bandwith and security may be more relevant than the cost of equipment when evaluating the costs and cost-effectiveness of telemedicine.

There was some discordant findings in reviews. One review found that the average consultation time for telemedicine was longer than for a face-to-face consultation, whilst another review found that telemedicine consultations were quicker [75, 60]. This is an important consideration in the context of physician reimbursement and emphasizes the context specificity of cost-effectiveness studies, which varies depending on the accuracy of cost information, specialty area, setting, and health care systems. It is also important to consider how much societal value is placed on the improvement of a particular health status or population e.g. the cost-effectiveness threshold. In addition, as observed in one review, health care systems may consider equitable factors, such as initial additional costs for delivering healthcare to remote regions, to reduce health inequalities with potentially larger future cost savings [69]. These factors all limit the comparablilty and generalizability of findings and make it difficult to reach a robust conclusion of whether telemedicine services are cost-effective or not.

This umbrella review finds that patients demonstrate high acceptability and satisfaction with telemedicine. Telemedicine affords patients convenience, and independence to manage their conditions at home or within their communities. The cost of devices and technological illiteracy may present a barrier to patient uptake of telemedicine especially in low-income populations and low resource settings. Other identified patient barriers to the wider use of telemedicine associated with high dropout rates and attrition are modifiable from the implementers perspective, and include technical and usability challenges. This umbrella review also finds that telemedicine is feasible and identifies several factors that may affect its sustainability.

### Limitations

This review intended to provide a summary of the current evidence on telemedicine within the OECD, however results within this review are limited by several factors. First, by synthesizing evidence from reviews, and relying on review author interpretations, some important details that may have been included in primary studies was lost. Furthermore, a small number of primary studies (5%) from non-OECD countries were included in reviews. Second, the overall methodological quality of the included reviews was low. In particular, several economic evaluations were limited by poor reporting quality or unavailable data. Third, publication bias was noted in some reviews, meaning that positive results on the effect of telemedicine were more likely to be published. Fourth, language bias may have been introduced by the exclusion of non-English language studies. Finally, telemedicine is a rapidly evolving field, and although we attempted to include the most current review level evidence, many systematic reviews included much older primary studies. The inclusion of evidence from older primary studies may lead to conclusions regarding clinical and cost-effectiveness that are no longer timely. For example, the costs associated with telemedicine may be lower today compared to a decade ago due the lower costs of the technology that contribute to the ubiquitous use of smart phones, fitness monitors, and high-speed broadband. Improved clinical practice and revised guidelines within this period may also narrow the gap between the observed effect of telemedicine and face-to-face care. These factors may limit the generalizability of the evidence presented in this review.

## Conclusion

The literature on telemedicine is diverse and rapidly growing. Most of the included systematic reviews, and primary studies focus on the effectiveness of telemedicine interventions, however decision makers are also concerned with cost-effectiveness, patient-centredness and implementation. This umbrella review finds that telemedicine is comparable to face-to-face care across several disease and specialtiy areas. Patients find interventions acceptable and report high satisfaction levels, although barriers to uptake and use, such as lack of training and usability problems remain. The overall quality of the evidence included in this umbrella review was low. Future systematic reviews may be improved by reporting protocols, including a list of excluded full-text studies, and sources of funding of primary studies in supporting materials. Interventions should be clearly defined, and stratifed by the type of telemedicine used (e.g remote monitoring vs. video consultation) to provide results that are more useful for practice, research and decision making.

The current evidence on cost-effectiveness is limited, both in availability and generalizability. Policy intervention may be required through regulation to ensure that DTC telemedicine interventions are user-friendly, clinically beneficial, and can integrate into existing health care systems. Health care reimbursement models may need to be adapted, acknowledging that telemedicine may take as much time as face-to-face care. Initial set-up costs could be an important barrier to telemedicine uptake, although these costs are likely to be offset in the medium to long-term. All these factors are important if telemedicine interventions are to deliver person-centred high-quality cost-effective care.

The provision of health care for sicker patients and those who live in rural areas is also relevant in the context of telemedicine. There is often a trade-off between efficiency and equity when delivering care to remote populations. An unintended consequence of telemedicine is that it is likely to reach relatively healthier and tech savvy patients in urban areas, rather than sicker digitally excluded patients in rural areas. Again, policy oversight may be required to improve access to health care, and to ensure that telemedicine is inclusive in reaching those patients who already experience limitations in accessing conventional models of care.

## Supporting information

S1 File Search strategy and reviews excluded at full text stage. (DOCX)

S2 File Characteristics of included systematic reviews. (DOCX)

S3 File Methodological quality of included systematic reviews. (DOCX)
