# Supplementary material for: Telemedicine in the OECD: An umbrella review of clinical and cost-effectiveness, patient experience and implementation
Source: PLoS One. 2020 Aug 13;15(8):e0237585. doi: 10.1371/journal.pone.0237585 (PMC7425977; doi:10.1371/journal.pone.0237585)
Supplement: S1 File — (DOCX) [file pone.0237585.s001.docx]

# Search Strategy for PubMed/Medline

PUBMED SEARCH

| 1 | OECD 36 | 12,597,376 |
| --- | --- | --- |
| 2 | 1 and Mesh terms [telemedicine] | 16142 |
| 3 | 2 and Systematic review (article type) | 389 |
| 4 | 3 and Date of publication (01/01/2014 – 01/02/2019) | **268** |

MEDLINE COMPLETE

| 1 | OECD 36 | 13,524,017 |
| --- | --- | --- |
| 2 | MESH TERMS Telemedicine OR remote consultation | 12,471 |
| 3 | S1 and S2 | 6,157 |
| 4 | S3 and (SU subject terms) systematic review or meta-analysis | 54 |
| 5 | S4 and published date (0/01/2014 to 01/02/2019) | **39** |

# OECD Countries^[[1]](#footnote-1)^

1. Australia
2. Austria
3. Belgium
4. Canada
5. Chile
6. Czech Republic
7. Denmark
8. Estonia
9. Finland
10. France
11. Germany
12. Greece
13. Hungary
14. Iceland
15. Ireland
16. Israel
17. Italy
18. Japan
19. Korea
20. Latvia
21. Lithuania
22. Luxembourg
23. Mexico
24. Netherlands
25. New Zealand
26. Norway
27. Poland
28. Portugal
29. Slovak Republic
30. Slovenia
31. Spain
32. Sweden
33. Switzerland
34. Turkey
35. United Kingdom
36. United States

# Reviews excluded at full text stage and reasons for exclusion

|  | **Review Citation** | **Reason for Exclusion** |
| --- | --- | --- |
| [1] | Abedtash H, Holden RJ. Systematic review of the effectiveness of health-related behavioral interventions using portable activity sensing devices (PASDs). J Am Med Informatics Assoc JAMIA 2017;24:1002–13. https://doi.org/10.1093/jamia/ocx006. | Effectiveness: no meta-analysis |
| [2] | AlDossary S, Martin-Khan MG, Bradford NK, Smith AC. A systematic review of the methodologies used to evaluate telemedicine service initiatives in hospital facilities. Int J Med Inform 2017;97:171–94. https://doi.org/10.1016/j.ijmedinf.2016.10.012. | Outcomes not relevant to review outcome |
| [3] | Amatya B, Galea MP, Kesselring J, Khan F. Effectiveness of telerehabilitation interventions in persons with multiple sclerosis: A systematic review. Mult Scler Relat Disord 2015;4:358–69. https://doi.org/10.1016/j.msard.2015.06.011. | Effectiveness: no meta-analysis |
| [4] | Avgousti S, Christoforou EG, Panayides AS, Voskarides S, Novales C, Nouaille L, et al. Medical telerobotic systems: current status and future trends. Biomed Eng Online 2016;15:96. https://doi.org/10.1186/s12938-016-0217-7. | Outcomes not relevant to review outcome |
| [5] | Ballini L, Negro A, Maltoni S, Vignatelli L, Flodgren G, Simera I, et al. Interventions to reduce waiting times for elective procedures. Cochrane Database Syst Rev 2015:CD005610. https://doi.org/10.1002/14651858.CD005610.pub2. | Effectiveness: no meta-analysis |
| [6] | Band R, Bradbury K, Morton K, May C, Michie S, Mair FS, et al. Intervention planning for a digital intervention for self-management of hypertension: a theory-, evidence- and person-based approach. Implement Sci IS 2017;12:25. https://doi.org/10.1186/s13012-017-0553-4. | Outcomes not relevant to review outcome |
| [7] | Buekers J, De Boever P, Vaes AW, Aerts J-M, Wouters EFM, Spruit MA, et al. Oxygen saturation measurements in telemonitoring of patients with COPD: a systematic review. Expert Rev Respir Med 2018;12:113–23. https://doi.org/10.1080/17476348.2018.1417842. | Not telemedicne |
| [8] | Clark RA. Telehealth in the Elderly with Chronic Heart Failure: What Is the Evidence? Stud Health Technol Inform 2018;246:18–23. | Not systematic review |
| [9] | Conway A, Inglis SC, Clark RA. Effective technologies for noninvasive remote monitoring in heart failure. Telemed J E-Health Off J Am Telemed Assoc 2014;20:531–8. https://doi.org/10.1089/tmj.2013.0267. | Not systematic review |
| [10] | Davies EB, Morriss R, Glazebrook C. Computer-delivered and web-based interventions to improve depression, anxiety, and psychological well-being of university students: a systematic review and meta-analysis. J Med Internet Res 2014;16:e130. https://doi.org/10.2196/jmir.3142. | Not telemedicne |
| [11] | de la Torre Díez I, Alonso SG, Hamrioui S, López-Coronado M, Cruz EM. Systematic Review about QoS and QoE in Telemedicine and eHealth Services and Applications. J Med Syst 2018;42:182. https://doi.org/10.1007/s10916-018-1040-4. | Outcomes not relevant to review outcome |
| [12] | de Ridder M, Kim J, Jing Y, Khadra M, Nanan R, M. de R, et al. A systematic review on incentive-driven mobile health technology: As used in diabetes management. J Telemed Telecare 2017;23:26–35. https://doi.org/10.1177/1357633X15625539. | Outcomes not relevant to review outcome |
| [13] | Donoghue K, Patton R, Phillips T, Deluca P, Drummond C. The effectiveness of electronic screening and brief intervention for reducing levels of alcohol consumption: a systematic review and meta-analysis. J Med Internet Res 2014;16:e142. https://doi.org/10.2196/jmir.3193. | Outcomes not relevant to review outcome |
| [14] | Elbert NJ, van Os-Medendorp H, van Renselaar W, Ekeland AG, Hakkaart-van Roijen L, Raat H, et al. Effectiveness and cost-effectiveness of ehealth interventions in somatic diseases: a systematic review of systematic reviews and meta-analyses. J Med Internet Res 2014;16:e110. https://doi.org/10.2196/jmir.2790. | Not systematic review |
| [15] | Fernández Aranda MI. Obstáculos para la implantación de la telemedicina en el ámbito de Obstetricia. Metas de Enfermería 2018;21:50–6. | Non-english language |
| [16] | Finnane A, Dallest K, Janda M, Soyer HP. Teledermatology for the Diagnosis and Management of Skin Cancer: A Systematic Review. JAMA Dermatology 2017;153:319–27. https://doi.org/10.1001/jamadermatol.2016.4361. | Effectiveness: no meta-analysis |
| [17] | Fitzner KK, Heckinger E, Tulas KM, Specker J, McKoy J, K.K. F, et al. Telehealth technologies: changing the way we deliver efficacious and cost-effective diabetes self-management education. J Health Care Poor Underserved 2014;25:1853–97. https://doi.org/10.1353/hpu.2014.0157. | Effectiveness: no meta-analysis |
| [18] | Gandhi S, Chen S, Hong L, Sun K, Gong E, Li C, et al. Effect of Mobile Health Interventions on the Secondary Prevention of Cardiovascular Disease: Systematic Review and Meta-analysis. Can J Cardiol 2017;33:219–31. https://doi.org/10.1016/j.cjca.2016.08.017. | Outcomes not relevant to review outcome |
| [19] | Hamine S, Gerth-Guyette E, Faulx D, Green BB, Ginsburg AS. Impact of mHealth Chronic Disease Management on Treatment Adherence and Patient Outcomes: A Systematic Review. J Med Internet Res 2015;17:e52. https://doi.org/10.2196/jmir.3951. | Outcomes not relevant to review outcome |
| [20] | Inglis SC, Conway A, Cleland JG, Clark RA. Is age a factor in the success or failure of remote monitoring in heart failure? Telemonitoring and structured telephone support in elderly heart failure patients. Eur J Cardiovasc Nurs J Work Gr Cardiovasc Nurs Eur Soc Cardiol 2015;14:248–55. https://doi.org/10.1177/1474515114530611. | Not systematic review |
| [21] | Jung Y, Kim J, Park DA. [Effectiveness of Telemonitoring Intervention in Children and Adolescents with Asthma: A Systematic Review and Meta-Analysis]. J Korean Acad Nurs 2018;48:389–406. https://doi.org/10.4040/jkan.2018.48.4.389. | Non-english language |
| [22] | Karlsen C, Ludvigsen MS, Moe CE, Haraldstad K, Thygesen E. Experiences of community-dwelling older adults with the use of telecare in home care services: a qualitative systematic review. JBI Database Syst Rev Implement Reports 2017;15:2913–80. https://doi.org/10.11124/JBISRIR-2017-003345. | Interventions not telemedicine e.g. personal alarms, fall dectectors |
| [23] | Khan F, Amatya B, Kesselring J, Galea M. Telerehabilitation for persons with multiple sclerosis. Cochrane Database Syst Rev 2015:CD010508. https://doi.org/10.1002/14651858.CD010508.pub2. | Effectiveness: no meta-analysis |
| [24] | Kitsiou S, Paré G, Jaana M. Effects of home telemonitoring interventions on patients with chronic heart failure: an overview of systematic reviews. J Med Internet Res 2015;17:e63. https://doi.org/10.2196/jmir.4174. | Not systematic review |
| [25] | Leavey K, Hawkins R. Is cognitive behavioural therapy effective in reducing suicidal ideation and behaviour when delivered face-to-face or via e-health? A systematic review and meta-analysis. Cogn Behav Ther 2017;46:353–74. https://doi.org/10.1080/16506073.2017.1332095. | Effectiveness: no meta-analysis |
| [26] | Loucas CE, Fairburn CG, Whittington C, Pennant ME, Stockton S, Kendall T. E-therapy in the treatment and prevention of eating disorders: A systematic review and meta-analysis. Behav Res Ther 2014;63:122–31. https://doi.org/10.1016/j.brat.2014.09.011. | Effectiveness: no meta-analysis |
| [27] | Mackintosh N, Terblanche M, Maharaj R, Xyrichis A, Franklin K, Keddie J, et al. Telemedicine with clinical decision support for critical care: a systematic review. Syst Rev 2016;5:176. https://doi.org/10.1186/s13643-016-0357-7. | Effectiveness: no meta-analysis |
| [28] | Mistry H, Garnvwa H, Oppong R. Critical appraisal of published systematic reviews assessing the cost-effectiveness of telemedicine studies. Telemed J E-Health Off J Am Telemed Assoc 2014;20:609–18. https://doi.org/10.1089/tmj.2013.0259. | Outcomes not relevant to review outcome |
| [29] | Morrison D, Wyke S, Agur K, Cameron EJ, Docking RI, Mackenzie AM, et al. Digital asthma self-management interventions: a systematic review. J Med Internet Res 2014;16:e51. https://doi.org/10.2196/jmir.2814. | Effectiveness: no meta-analysis |
| [30] | Mueller KJ, Potter AJ, MacKinney AC, Ward MM. Lessons from tele-emergency: improving care quality and health outcomes by expanding support for rural care systems. Health Aff (Millwood) 2014;33:228–34. https://doi.org/10.1377/hlthaff.2013.1016. | Not systematic review |
| [31] | Murphy LA, Harrington P, Taylor SJ, Teljeur C, Smith SM, Pinnock H, et al. Clinical-effectiveness of self-management interventions in chronic obstructive pulmonary disease: An overview of reviews. Chron Respir Dis 2017;14:276–88. https://doi.org/10.1177/1479972316687208. | Effectiveness: no meta-analysis |
| [32] | Orton M, Agarwal S, Muhoza P, Vasudevan L, Vu A. Strengthening Delivery of Health Services Using Digital Devices. Glob Heal Sci Pract 2018;6:S61–71. https://doi.org/10.9745/GHSP-D-18-00229. | Not systematic review |
| [33] | Piga M, Cangemi I, Mathieu A, Cauli A. Telemedicine for patients with rheumatic diseases: Systematic review and proposal for research agenda. Semin Arthritis Rheum 2017;47:121–8. https://doi.org/10.1016/j.semarthrit.2017.03.014. | Effectiveness: no meta-analysis |
| [34] | Rathbone AL, Prescott J. The Use of Mobile Apps and SMS Messaging as Physical and Mental Health Interventions: Systematic Review. J Med Internet Res 2017;19:e295. https://doi.org/10.2196/jmir.7740. | Effectiveness: no meta-analysis |
| [35] | Rintala A, Hakala S, Paltamaa J, Heinonen A, Karvanen J, Sjögren T. Effectiveness of technology-based distance physical rehabilitation interventions on physical activity and walking in multiple sclerosis: a systematic review and meta-analysis of randomized controlled trials. Disabil Rehabil 2018;40:373–87. https://doi.org/10.1080/09638288.2016.1260649. | Interventions not telemedicine e.g.nintendo wii, tailored home based activities |
| [36] | Rose T, Barker M, Maria Jacob C, Morrison L, Lawrence W, Strömmer S, et al. A Systematic Review of Digital Interventions for Improving the Diet and Physical Activity Behaviors of Adolescents. J Adolesc Heal Off Publ Soc Adolesc Med 2017;61:669–77. https://doi.org/10.1016/j.jadohealth.2017.05.024. | Effectiveness: no meta-analysis |
| [37] | Ross J, Stevenson F, Lau R, Murray E. Factors that influence the implementation of e-health: a systematic review of systematic reviews (an update). Implement Sci IS 2016;11:146. https://doi.org/10.1186/s13012-016-0510-7. | Not systematic review |
| [38] | Schnall R, Travers J, Rojas M, Carballo-Diéguez A. eHealth interventions for HIV prevention in high-risk men who have sex with men: a systematic review. J Med Internet Res 2014;16:e134. https://doi.org/10.2196/jmir.3393. | Effectiveness: no meta-analysis |
| [39] | Tran V, Lam MK, Amon KL, Brunner M, Hines M, Penman M, et al. Interdisciplinary eHealth for the care of people living with traumatic brain injury: A systematic review. Brain Inj 2017;31:1701–10. https://doi.org/10.1080/02699052.2017.1387932. | Outcomes not relevant to review outcome |
| [40] | Vallury KD, Jones M, Oosterbroek C. Computerized Cognitive Behavior Therapy for Anxiety and Depression in Rural Areas: A Systematic Review. J Med Internet Res 2015;17:e139. https://doi.org/10.2196/jmir.4145. | Effectiveness: no meta-analysis |
| [41] | Yusif S, Hafeez-Baig A, Soar J. e-Health readiness assessment factors and measuring tools: A systematic review. Int J Med Inform 2017;107:56–64. https://doi.org/10.1016/j.ijmedinf.2017.08.006. | Outcomes not relevant to review outcome |

1. Membership on 01 Feb 2019 [↑](#footnote-ref-1)
