# Supplementary material for: Telemedicine in the OECD: An umbrella review of clinical and cost-effectiveness, patient experience and implementation
Source: PLoS One. 2020 Aug 13;15(8):e0237585. doi: 10.1371/journal.pone.0237585 (PMC7425977; doi:10.1371/journal.pone.0237585)
Supplement: S2 File — (DOCX) [file pone.0237585.s002.docx]

# Table 1: Characteristics of Clinical-effectiveness Reviews

| **First Author (Year)** | **Included studies (n)** | **Objective** | **Intervention** | **Comparator** | **Outcomes** | **Findings and authors conclusions** | | **AMSTAR Review Quality** |
| --- | --- | --- | --- | --- | --- | --- | --- | --- |
| Adamse (2018) | 16 | To review the effectiveness of TM on chronic pain, compared to or in addition to TAU. | i. Exercise based TM in addition to TAU or no intervention | i. No intervention ii. TAU | i. Pain ii. Physical activity iii. Activities of daily living (ADL) iv. Quality of Life (QoL) | TM compared to no intervention was effective in reducing pain (MD −0.57, 95% CI −0.81; −0.34) and showed no difference compared to usual care for ADL (SMD 0.08, 95% CI -0.37; 0.53). TM maybe an effective substitution for TAU, but the quality of evidence is limited. | Critically Low | |
| Agostini (2015) | 12 | To determine if TR was more effective than TAU to regain motor function, in different populations of patients. | i. Telemonitored exercise training ii. self-monitoring iii. pedometer | i. TAU | i. Motor function ii. Upper extremity function iii. Mobility iv. Independence | TR effective for cardiac (SMD = 0.24, CI 95% = 0.04, 0.43) and total knee atrophy (TKA) patients (MD = −5.17, CI 95% = −9.79, −0.55), but inconclusive for neurological patients (SMD = 0.08, CI 95% = −0.13, 0.29) | Low | |
| Cottrell (2017) | 16 | To evaluate the effectiveness of treatment delivered via real-time TR for to manage musculoskeletal conditions, and determine if real-time TR is comparable to TAU | i Real-time TR via video conferencing and telephone | i. F2F ii. TAU | i. Pain ii. Physical function (PF) | Real-time TR is effective for the improvement of PF (SMD 1.63, 95%CI 0.92-2.33). In sub-group analyses TR in addition to TAU was more effective (SMD 0.64, 95%CI 0.43-0.85, I2=10%) than TAU alone. TR alone is equivalent to F2F care (SMD MD 0.14, 95% CI −0.10–0.37, I2 = 0%) for the improvement of PF. Pain improvement was comparable between cohorts (SMD 0.66, 95%CI −0.27–1.60, I2=96%) post intervention. | Critically Low | |
| Cruz (2014) | 9 | To evaluate the effectiveness of home-based telemonitoring (HTM) to reduce healthcare utilization and improve health‐related outcomes of patients with COPD. | i. Home-based telemonitoring | i. F2F ii. TAU | i. Hospitalization rates ii. Length of hospital stay iii. Emergency department visit rates iv. HRQoL | HTM seems effective in reducing hospitalization rates, (RR = 0.72; 95% CI= 0.53–0.98; p=0.034), and improves health-related outcomes for COPD patients; but no differences in the other healthcare utilization outcomes were observed. | Critically Low | |
| Dario (2017) | 11 | To evaluate whether telehealth interventions improve pain, disability, function, and quality of life in non-specific lower-back pain (LBP). | i. telemonitoring (pedometer) ii. web-based programs iii. online support groups iv. telephone interventions | i. TAU | i. Pain reduction ii. Reduce disability iii. Improve physical function iv. Quality of life | In chronic LBP, telehealth interventions had no significant effect on pain at short-term follow-up (n=4: 1,089 participants, WMD −2.61 95% CI −5.23 to 0.01) or medium-term follow-up (n=2: 441 participants, WMD: −0.94 points, 95% CI: −6.71 to 4.84) compared with a control group. Interventions combining telehealth and TAU were more beneficial than TAU alone in people with recent onset of LBP symptoms. | Low | |
| Deady et al 2017 | 10 | To review and evaluate the effects of eHealth prevention interventions for anxiety and depression. | i. telemonitoring ii. real-time iii. e-couch iv. web based v. email support vi. hybrid | i. TAU | i. Symptom reduction ii. Depression Prevention | MD between groups was 0.25 (95% CI:0.09, 0.41; p = 0.003) for depression outcome studies and 0.31 (95% CI: 0.10, 0.52; p = 0.004) for anxiety outcome studies, indicating a small but positive effect of the eHealth interventions. However, there is inadequate evidence on the medium to long-term effect of interventions to reduce the incidence of mental health disorders | Critically Low | |
| Direito et al 2017 | 21 | To compare the effectiveness of mHealth interventions to promote physical activity (PA) and reduce sedentary behavior (SB) in young people and adults with a comparator exposed to TAU/minimal intervention | i. telemonitoring ii. hybrid | i. TAU | i. moderate to vigorous physical activity (MVPA) ii. walking iii. sedentary behavior iv. behavior change techniques | SB decreased following interventions compared to TAU (SMD −0.26, 95%CI −0.53, −0.00). Effects across studies were small to moderate and non-significant for total PA (SMD 0.14, 95 % CI −0.12, 0.41); MVPA (SMD 0.37, 95 % CI −0.03 to 0.77); and walking (SMD 0.14, 95 % CI −0.01 to 0.29). | Low | |
| Feltner et al 2014 | 47 | To assess the efficacy, comparative effectiveness, and harms of transitional care interventions to reduce readmission and mortality rates for adults hospitalized with heart failure (HF). | i. Telemonitoring (TM) ii. Real-time: structured telephone support (STS) | TAU | i. All-cause readmissions ii. Mortality iii. Heart failure specific readmissions | TM did not reduce all-cause readmission 3 trials (n=434) RR 1.11, CI (0.87,1.42), or mortality 3 trials (n=564) RR 0.93CI (0.25-3.48) rates. STS interventions reduced HF-specific readmission in one high-quality trial (n=182) RR 1.70 CI (0.82-3.51) but not all-cause readmissions 3 trials (n=434) RR 1.11 (0.87-1.42). Although STS reduced HF-specific readmission and mortality, TM is ineffective in reducing risk of readmission and mortality in HF patients. | Low | |
| Flodgren et al 2015 | 93 | To assess the effectiveness, acceptability and costs of interactive TM as an alternative to, or in addition to, TAU. | i. Real-time ii. Telemonitoring iii. Hybrid | i. F2F or TAU | i. Mortality ii. Quality of life iii. Costs iv. Clinical outcomes v. Effect of treatment | In cardiac patients, TM did not reduce all‐cause mortality (16 studies; n=5239; RR:0.89, 95% CI 0.76,1.03, P = 0.12; I2=44%) at six months follow‐up. In diabetic patients (16 studies; n=2768) lower HbA1c levels were observed in those allocated to TM than in controls (MD ‐0.31, 95% CI ‐0.37‐0.24; P< 0.001; I2=42%, P=0.04) at 9 months follow‐up. TM in the management of heart failure appears to lead to similar health outcomes as F2F care and can improve the control of blood glucose in those with diabetes. | High | |
| Hakala et al 2017 | 8 | To examine whether a technology-based distance intervention (TBI) promoting physical activity is more effective than a physical activity intervention without the use of technology. | i. Telemonitoring | i. TAU ii. Minimal Interventions | i. Physical activity levels | TBI were 12% more effective than TAU or minimal control interventions in increasing physical activity (RR:1.12; 95% CI:1.01,1.25, P=0.03). Compared to minimal control interventions, TBI were 19% more effective (RR: 1.19; 95% CI 1.05 to 1.35, P=0.0096). In the interventions targeting patients, use of technology was 25% more effective than non-use (P=0.027). TBI are more effective than TAU in promoting physical activity, especially when interventions are targeted to patients. | Low | |
| Huang et al 2015 | 9 | To determine the effectiveness of telehealth intervention delivered cardiac rehabilitation (CR) compared with center-based supervised CR. | i. Home-based telemonitoring ii. Telephone support | i. Centre based programs (F2F) | i. All-cause mortality ii. Weight iii. Blood pressure iv. HRQoL | No difference was found between telehealth interventions and center-based CR in exercise capacity SMD –0.01; 95% CI –0.12–0.10), weight (SMD –0.13; 95% CI –0.30–0.05), systolic and diastolic blood pressure(MD –1.27; 95% CI –3.67–1.13 and MD 1.00; 95% CI –0.42–2.43, respectively), mortality (RR 1.15; 95% CI 0.61–2.19), quality of life and psychosocial state. CR delivered through telehealth interventions have similar outcomes compared to center-based supervised program in low to moderate risk patients. | Critically Low | |
| Huang et al 2014 | 6 | To review the effectiveness of distance management methods in the management of adult inflammatory bowel disease (IBD) patients. | i. Telemonitoring ii. Hybrid | I. F2F or TAU | i. QoL ii.. Relapse rate iii. No of clinic visits | TM (3 studies, n=1463) interventions improved quality of life in distance management patients, IBD QoL score 7.28 (95%CI: -3.25-17.81) points higher than standard clinic follow-up. There was a significant decrease in the clinic visit rate among distance management patients mean difference -1.08 (95%CI: -1.60--0.55), but no significant change in relapse rate or hospital admission rate. Distance management of IBD decreases clinic visit utilization, but it does not significantly affect relapse, or hospital admission rates. | High | |
| Huang et al 2015 | 18 | To review the published literature on the effects of telecare intervention in patients with type 2 diabetes and inadequate glycemic control. | i. Remote self-monitoring | i. F2F consultations | i. HbA1C ii. BMI iii. Hypoglycemic events | TM significantly improved glycemic control of patients with Type 2 diabetes compared to the group receiving standard care, mean HbA1c values were reduced by −0.54 (95% CI, −0.75, −0.34; P<0.05). No significant difference was found for adverse events and BMI between TM and control groups | Critically Low | |
| Hui et al 2017 | 12 | To identify mobile app features that aid self-management of asthma and are associated with adoption, adherence, and effectiveness. | i. Telemonitoring ii. real-time iii. hybrid | i. No intervention or TAU | i. Asthma control ii. Acute exacerbations | Meta-analysis n= 3. TM was effective, compared to no intervention or TAU in improving asthma control (mean difference −0.25 [95% CI, −0.37 to −0.12]). | Critically Low | |
| Hutchesson et al 2015 | 84 | To evaluate the effectiveness of eHealth interventions for the prevention and treatment of overweight and obesity in adults. | i. Telemonitoring | i. TAU or minimal intervention (MI) | i. Weight loss ii. weight loss maintenance iii. weight gain prevention iv. weight loss and maintenance | TM groups demonstrated significantly greater weight loss (kg) compared with control (MD−2.70 [−3.33,−2.08], P < 0.001) or MI (MD −1.40 [−1.98,−0.82], P < 0.001), and in eHealth weight loss interventions with behavioural components or extra technologies (MD 1.46 [0.80, 2.13], P < 0.001) compared with standard eHealth program. eHealth interventions are a treatment option for obesity. However, there is insufficient evidence for the effectiveness of eHealth interventions for weight loss maintenance or weight gain prevention. | Low | |
| Jeon et al 2015 | 37 | To review the effect of mobile technology-based interventions in nursing | i. Telemonitoring using mobile technology | i. No intervention or TAU | i. Feasibility ii. Weight reduction iii. Fasting plasma glucose iv. health behaviour change | TM intervention had a slightly positive effect on weight reduction (Hedges’ g: -0.23, 95% CI: -0.43 to -0.03), and fasting plasma glucose (Hedges’ g: -0.35, 95% CI: -0.54 to -0.16) | Critically Low | |
| Joiner et al 2017 | 22 | To describe Diabetes Prevention Program (DPP)-based lifestyle interventions delivered via electronic, mobile, eHealth interventions and estimate the effect on weight loss. | i. Telemonitoring ii. Telemonitoring with remote behavioural support. | i. F2F DPP interventions | Weight loss | The overall estimate across all the DPP-based eHealth interventions of on mean percentage weight change was − 3.98% (95% CI of − 4.49,− 3.46; I2 = 88.2%). The subtotal estimate across the interventions (n=9) with remote behavioral support was − 4.31% (95% CI− 5.26,− 3.37; I2 = 78.4%), and the subtotal estimate across interventions with F2F behavioral support was − 4.65% (95% CI of − 6.63, − 2.67; I2 = 94.5%). There is promising evidence of the efficacy of DPP-based eHealth interventions on weight loss. | Critically Low | |
| Kelly et al 2016 | 25 | To assess the effectiveness of telehealth dietary interventions (TDI) at facilitating dietary change in chronic disease | i. Video-conults ii. Telemonitoring iii. Hybrid | i. TAU or no intervention | i. Weight ii. BMI iii. Blood pressure iv. Serum lipids and glycated hemoglobin | TDIs were effective at improving diet quality [SMD: 0.22 (95% CI: 0.09, 0.34), P = 0.0007], fruit and vegetable intake [MD 1.04 servings/d (95% CI: 0.46, 1.62 servings/d), P = 0.0004] | Low | |
| Kepplinger et al 2016 | 7 | To evaluate the safety and efficacy of IV thrombolysis (IVT) with tissue plasminogen activator (tPA) delivered through telestroke networks in patients with acute ischemic stroke | i. Real-time based on remote clinical and technological evaluation via audio visual video communication | i. TAU i.e. patients treated at stroke center | i. Safety ii. Efficacy | Thrombolysis was largely restricted to the 3-hour time window. Symptomatic intracerebral hemorrhage rates were similar between patients subjected to TM-guided IVT and those receiving tPA at stroke centers (RR = 1.01, 95% CI 0.37–2.80; p = 0.978) with low evidence of heterogeneity (I2 = 37%; p = 0.189). There was no difference in mortality (RR = 1.04, 95% CI 0.74–1.48; p = 0.806) or in functional independence (RR = 1.11, 95% CI 0.78–1.57; p = 0.565) at 3 months between TM-guided and stroke center thrombolysis. | Low | |
| Klersy et al 2016 | 13 | To determine whether device telemonitoring (DTM) reduces healthcare utilization over standard of care (SoC), without compromising patient outcomes. | i. Telemonitoring | i. F2F or SoC i. Hybrid | i. Cardiac hospitalizations ii. Unplanned Emergency Room (ER) visits or cardiac hospitalizations iii. Cardiac death iv. Death | DTM was associated with a reduction in total number of visits [planned, unplanned, and ER] [RR 0.56; 95% (CI) 0.43–0.73, P < 0.001]. Rates of cardiac hospitalizations (RR 0.96; 95% CI 0.82–1.12, P = 0.60) and the composite endpoints of ER, unplanned hospital visits, or hospitalizations (RR 0.99; 95% CI 0.68–1.43, P = 0.96) was similar between the DTM and the SoC groups. In RCTs, DTM is associated with a marked reduction in planned hospital visits and overall costs, without compromising survival or markedly increasing unplanned hospital visits, ER visits, or hospitalizations. | High | |
| Kotb et al 2015 | 30 | To determine the comparative impact of different TM options for a specific population such as individuals with heart failure (HF). | i. Telemonitoring ii. Telemonitoring with structured telephone support (STS) | i. TAU or F2F ii. hybrid | i. All-cause mortality ii. All cause hospitalization iii. Hospitalization due to heart failure | Compared to TAU, STS reduced the odds of mortality (OR 0.80; 95% CrI [0.66 to 0.96]) and hospitalizations due to HF (0.69; [0.56 to 0.85]). Telemonitoring also reduced the odds of mortality (0.53; [0.36 to 0.80]) and hospitalizations related to HF (0.64; [0.39 to 0.95]) compared to usual post-discharge care. Compared to TAU, STS and telemonitoring significantly reduced the odds of deaths and hospitalization due to heart failure. | Critically Low | |
| Lee et al 2016 | 3 | To review the effectiveness of pediatric obesity intervention studies using mobile technology. | i. Telemonitoring mobile interventions e.g. apps ii. Hybrid | i. TAU or no intervention | i. BMI ii. Daily exercise iii. intake of sugar-sweetened beverage (SSBs) | Mobile interventions had no significant effect on BMI (g: -0.073, 95% CI: -0.031 to 0.185), or daily exercise and SSB intake; (Hedges’ g: 0.189, 95% CI: -0.355 to 0.733; Hedges’ g: -0.316, 95% CI: -0.764 to 0.131). | Critically Low | |
| Lee et al 2018 | 7 | To review the effectiveness of telehealth interventions on improving oral anticoagulation management | i. Real-time: telephone communication between provider and patient | i. In person visits: TAU | i. Major bleeding ii. Any bleeding | (3 Meta-analysis n = 6955) showed significant improvements in the telehealth group for major thromboembolic events (RR 0.43, 95% CI 0.25–0.74, p = 0.002), but no significant difference for major bleeding events (RR 0.83, 95% CI 0.52–1.33, p = 0.44). Telehealth interventions may lower the risk of major thromboembolic events, but not other clinically important outcomes. | Critically Low | |
| Linde et al 2015 | 30 | To review whether psychological treatments are effective for treating depressed primary care patients in comparison with TAU or placebo, taking the type of therapy and its delivery mode into account. | i. Real-time cognitive behavioural therapy (CBT) ii. remote therapist led i. hybrid | i. F2F, TAU or no intervention | i. Depression scores | Compared with control, SMD was −0.30 (95% CI, −0.48, −0.13) for F2F CBT, −0.14 (−0.40, 0.12) for F2F problem-solving therapy, −0.24 (−0.47 to −0.02) for F2F interpersonal psychotherapy, −0.28 (−0.44 to −0.12) for other F2F psychological interventions, −0.43 (−0.62 to −0.24) for remote therapist-led CBT, −0.56 (−1.57 to 0.45) for remote therapist-led problem-solving therapy, −0.40 (−0.69 to −0.11) for guided self-help CBT, and −0.27 (−0.44 to −0.10) for no or minimal contact CBT. Remote CBT interventions seem to yield effects similar to face to face interventions. | Low | |
| Liu et al 2017 | 13 | To examine the potential role of mHealth on vascular risk factor control, including diabetes mellitus, hypertension, hyperlipidemia, and smoking. | i. Telemonitoring: mHealth interventions | i. TAU or no intervention | i. Glycemic control ii. Smoking cessation | mHealth resulted in greater Hemoglobin A1c reduction at 6 months (6 studies, n= 663, SMD: −0.44; 95% CI: [−0.82, −0.06], P = 0.02; MD of decrease in HbA1c: −0.39%; 95% CI: [−0.74, −0.04], P = 0.03). mHealth also lead to relatively higher smoking abstinence rates at 6 months (7 studies, n=9514; OR: 1.54; 95% CI: [1.24, 1.90], P < 0.0001). | Critically Low | |
| Lundell et al 2015 | 9 | To investigate the effects of telehealth care on physical activity level, physical capacity and dyspnea in patients with COPD, and to describe the interventions used. | i. Home-based monitoring ii. Weekly phone call | i. TAU | i. Physical activity level (PAL) ii. Physical capacity (PC) iii. Dyspnea | For PAL, there was a significant effect favoring telehealth care (MD, 64.7 min; 95% CI, 54.4–74.9). No difference between groups was found for PC (MD, −1.3 m; 95% CI, −8.1–5.5) and dyspnea (SMD, 0.088; 95% CI, −0.056–0.233). The use of telehealth care may lead to improvements in PAL, although the results should be interpreted with caution given the heterogeneity in studies. | Low | |
| Marx et al 2018 | 9 | To determine the efficacy of telehealth methods in delivering malnutrition-related interventions to community-dwelling older adults. | i. Real time (telephone consultation with dietitian or dietetic assistant) ii. Telemonitoring | i. TAU | i. Nutrition status ii. Feasibility iii. Effectiveness iv. Body weight v. Quality of life | Malnutrition-focused telehealth interventions improved protein intake in older adults by 0.13 g/kg body weight per day ([95%CI: 0.01–0.25]; P = .03; 2 studies; n = 200 I2 = 41%) and to improve quality of life (SMD: 0.55 [95%CI: 0.11–0.99]; P = .01; 4 studies, n=248; I2 = 84%). Telehealth is an effective method to deliver malnutrition-related interventions to older adults living at home, and is likely to result in clinical improvements compared with TAU or no intervention. | Low Quality | |
| McLean et al 2016 | 8 | To identify, summarise and synthesise the evidence for using interactive digital interventions to support patient self-management of asthma, and determine their impact. | i. Telemonitoring: self-monitoring | i. TAU | i. Change in clinical outcomes ii. Patient reported outcomes of wellbeing or quality of life | 3 meta-analysis n= 593. No significant differences and extremely high heterogeneity for Asthma Quality of Life (AQLQ) SMD 0.05; 95%(CI) 0.32, 0.22: I296.8) and asthma control (SMD 0.21; 95 % CI −0.05, .42; I2=87.4). Removal of the third study indicated significant improvement for both AQLQ (SMD 0.45; 95 % CI 0.13, 0.77: I2=0.34) and asthma control (SMD 0.54; 95 % CI 0.22, 0.86: I2=0.11). Digital self-management interventions for adults with asthma show promise, with some evidence of small beneficial effects on asthma control. | Low | |
| Merriel et al 2014 | 13 | To assess the effectiveness of telehealth interventions in the primary prevention of cardiovascular disease in adult patients in community settings. | i. Telemonitoring ii. real-time | i. F2F, TAU or self help | i. reducing systolic bp ii. Cholesterol iii. smoking status at baseline | 3 meta-analysis showed no clear evidence of reduction in overall risk of CVD (SMD − 0.37%, 95% CI − 2.08, 1.33). There was weak evidence for reduction in systolic blood pressure (SMD − 1.22 mm Hg 95% CI − 2.80, 0.35) and total cholesterol (SMD − 0.07 mmol/L 95% CI − 0.19, 0.06). There was no change in cholesterol or smoking rates. There is insufficient evidence to determine the effectiveness of telehealth interventions in reducing overall CVD risk. | Critically Low | |
| Ming et al 2016 | 7 | To determine whether TM solutions offer any advantages compared with the standard care for women with diabetes in pregnancy. | i. Telemonitoring | i. TAU | i. Maternal glycemic control | TM interventions showed a modest statistically significant improvement in HbA1c, mean HbA1c of women using TM was 5.33% (SD 0.70) compared with 5.45% (SD 0.58) in the TAU group; a mean difference of −0.12% (95% CI −0.23% to −0.02%). When this comparison was limited to women with gestational diabetes mellitus (GDM) only, the mean HbA1c of women using TM was 5.22% (SD 0.70) compared with 5.37% (SD 0.61) in the standard care group, mean difference −0.14% (95% CI −0.25% to −0.04%). There is insufficient evidence that TM is superior to TAU for women with diabetes in pregnancy; however, there was no evidence of harm. | Low | |
| Nair et al 2018 | 10 | To review the effectiveness of TM interventions to address maternal depression | i. telemonitoring ii. real-time (telephone) | i. waitlist ii. TAU iii. hybrid | i. Depression scores | 5 meta-analysis: (I2 = 74.2%; X2 = 14.63; df = 4; p =  0.06). Effect sizes ranged between −0.69 and −4.03. Interventions delivered via TM effectively alleviate maternal depression symptoms, improving treatment efficacy. | Low | |
| Oosterveenet al 2017 | 45 | To evaluate the effectiveness of eHealth behavioral interventions to improve smoking rates, nutrition behaviors, alcohol intake, physical activity levels and/or obesity (SNAPO) in young adults | i. Telemonitoring ii. Real-time (telephone counselling) | i. waitlist ii. no intervention iii. hybrid | i. Effectiveness | Significantly lower mean alcohol consumption/week in brief web or computer-based interventions compared to controls (MD − 2.43 [− 3.54, − 1.32], P < 0.0001, n = 10). There is some evidence for the short-term efficacy of eHealth SNAPO interventions for young adults, and for alcohol interventions but there is insufficient evidence for their efficacy in the longer-term, as well as which mode of delivery is most effective. | Low | |
| Raman et al 2017 | 11 | To compare the effects of different methods and settings for glucose monitoring for women with GDM on maternal and fetal, neonatal, child and adult outcomes, and use and costs of health care. | Home-monitoring: i. Remote monitoring | i. TAU | i. Pre-eclampsia ii. Pregnancy-induced hypertension iii. Caesarean section | TM versus standard care for glucose monitoring (5 studies), no clear differences between the TM and standard care groups for the mother: for pre‐eclampsia or pregnancy‐induced hypertension (RR) 1.49, 95% CI 0.69 to 3.20; n=275 4 studies ); caesarean section (RR 1.05, 95% CI 0.72 to 1.53; n=478, 5 studies). Evidence from 11 RCTs assessing different methods or settings for glucose monitoring for GDM suggests no clear differences for the primary outcomes or other secondary outcomes assessed in this review. | High | |
| Rasekaba et al 2015 | 4 | To evaluate the effect of TM on GDM service and maternal, and foetal outcomes. | i. Remote self-monitoring for gestational diabetes management | i. TAU ii. F2F GDMC (gestational diabetes management clinic) | i. Glycemic control ii. Blood glucose levels iii. Caesarean deliveries | TM intervention groups showed a SMD for glycaemic control -0.18 [-0.50, 0.14], (1-h and 2-h) post-prandial BGL -0.02[-0.36, 0.32], and caesarean deliveries 0.48 [0.10,2.35] compared to TAU. TM has the potential to streamline GDM service utilisation without compromising maternal and foetal outcomes. | High | |
| Rawstorn et al 2016 | 11 | To determine the benefits of telehealth exCR (exercise based cardiac rehabilitation) on exercise capacity and other modifiable cardiovascular risk factors compared with traditional exCR and TAU, among patients with coronary heart disease (CHD). | i. Telemonitoring ii. real-time TM | i. F2F or TAU (centre based) | i. Physical activity level ii. Maximal aerobic exercise capacity iii. Exercise adherence | Physical activity level was statistically significantly higher following telehealth exCR compared with centre-based exCR (SMD=9.84, 95% CI 8.05 to 11.64, and TAU (fixed effect SMD=0.29, 95% CI 0.07 to 0.50). Exercise adherence was statistically significantly higher following telehealth exCR (SMD=0.75, 95% CI 0.52 to 0.98). Telehealth exCR appears to be at least as effective as centre-based exCR for improving modifiable cardiovascular risk factors and functional capacity, and could enhance exCR utilisation by providing additional options for patients who cannot attend centre-based exCR. | Low | |
| Seiler et al 2017 | 15 | To evaluate existing eHealth/mHealth interventions developed to help manage cancer‐related fatigue (CRF); and summarize the best available evidence on their effectiveness. | i. Real-time ii. Online interventions iii. Remote monitoring iv. Smart phone apps | i. TAU ii. F2F iii. No intervention | i. Cancer-related fatigue ii. HRQoL | 9 meta‐analysis. eHealth studies revealed a statistically significant beneficial effect on CRF (r = .27, 95% CI [.1109 – .4218], P < 0.01). Therapist‐guided eHealth interventions were more effective then self‐guided interventions (r= .58, 95% CI: [.3136 – .5985, P < 0.001). Small to moderate effects were also observed for HRQoL (r = .17, 95% CI [.0384 – .3085], P < 0.05) and depression (r = .24, 95% CI [.1431 – .3334], P < 0.001). eHealth interventions appear to be effective for managing fatigue in cancer survivors with CRF. | Low | |
| Seyffert et al 2016 | 15 | To assess the effectiveness of internet-delivered cognitive behavioral therapy (iCBT) for insomnia. | i. Telemonitoring and real time iCBT | i. Waitlist | i. Sleep efficiency and severity of insomnia ii. Length of sleep iii. sleep quality iv. time in bed v. nocturnal awakenings | Sleep efficiency was 72% at baseline and improved by 7.2% (95% CI:5.1%, 9.3%; p<0.001) with internet-delivered cognitive behavioral therapy versus control. Internet-delivered CBT resulted in a decrease in the insomnia severity index by 4.3 points (95% CI: -7.1, -1.5; p = 0.017) compared to control. The severity of depression decreased by 2.3 points (95% CI: -2.9, -1.7; p = 0.013) in individuals who received internet-delivered cognitive behavioral therapy compared to control. There were no statistically significant differences between sleep efficiency, total sleep time, and insomnia severity index for internet-delivered versus in-person therapy with a trained therapist. | Critically Low | |
| Sherifali et al 2017 | 10 | To assess the effectiveness of eHealth technologies for weight management during pregnancy and the postpartum period and to review the efficacy of eHealth technologies on health behaviors, specifically nutrition and physical activity. | i. Remote monitoring ( web-based, email, personal digital assistant, handheld computer, home computer, or tablet app) | i. TAU or educational intervention | i. weight management in pregnant women or weight loss ii. in postpartum women iii. physical activity | In postpartum women, eHealth intervention resulted in a significant reduction in weight (−2.55 kg, 95% CI −3.81 to −1.28) after 3 to 12 months; but six studies found a nonsignificant reduction in weight gain for pregnant women (−1.62 kg, 95% CI −3.57 to 0.33) at approximately 40 weeks. This review found evidence for benefits of eHealth technologies on weight management in postpartum women only. | Critically Low | |
| Speyer et al 2018 | 43 | To describe telehealth interventions delivered by allied health professionals and nurses in rural and remote areas, and to compare the effects of telehealth interventions with standard face-toface interventions. | i. Video Consults ii. Telephone iii. internet based | i. TAU ii. real-time TM (telephone) | i. Effectiveness | 17 meta-analysis. No significant differences for interventions adopting a physical approach between telehealth and standard treatment (z (1) =0.335, p=0.737, g=0.178, 95% CI=–0.861–1.216). There were significant differences for interventions using a combination of cognitive and physical approaches between telehealth and standard treatment, with a moderate effect favouring telehealth-delivered interventions (z (7) =2.159, p=0.031, g=0.500, 95% CI=0.046–0.955). | Critically Low | |
| Stratton et al 2017 | 32 | To conduct a systematic review and meta-analysis evaluating the evidence for the effectiveness and examine the relative efficacy of different types of eHealth interventions for employees. | i. Telemonitoring: web-based CBT and mobile apps | i. TAU | i. Depression ii. Anxiety iii. Stress | 23 meta-analysis. eHealth interventions suggested a small positive effect at both post intervention (g = 0.24, 95% CI 0.13 to 0.35) and follow up (g = 0.23, 95% CI 0.03 to 0.42). The Stress Management interventions differed by whether delivered to universal or targeted groups with a moderately large effect size at both post-intervention (g = 0.64, 95% CI 0.54 to 0.85) and follow-up (g = 0.69, 95% CI 0.06 to 1.33) in targeted groups, but no effect in unselected groups. | Critically Low | |
| Su et al 2016 | 49 | To assess the overall effect of TM on diabetes management and to identify features of TM interventions that are associated with better diabetes management outcomes. | i. Telemonitoring ii. Real-time | i. TAU | i. Mean change in HbA1c | Results favored TM over conventional care (g = −0.48, p < 0.001) in diabetes management. The beneficial effect of TM was more pronounced among patients with type 2 diabetes (g = −0.63, p < 0.001) than among those with type 1 diabetes (g = −0.27, p = 0.027) (Q = 4.25, p = 0.04). | Critically Low | |
| Tchero et al 2017 | 10 | To evaluate whether TM can be effective in diabetic foot patient care. | i. Real-time TM (video consultation) | i. TAU | i. Healing time | 2 meta-analysis. TM and control groups had statistically similar healing time (43 vs 45 days; P = .83), healing time ratio adjusted for age (1 vs 1.4; P = .1), unhealed ulcers or loss to follow-up (3 of 20 vs 7 of 120; P = .13), and amputations (12 of 193 vs 14 of 182; P = .59). The odds of complete ulcer healing were statistically similar between the TM group and controls (OR= 0.86; 95% CI = 0.57-1.33; P = .53). | Critically Low | |
| Thabrew et al 2018 | 5 | To assess the effectiveness of e‐health interventions in comparison with attention placebos, psychological placebos, treatment as usual, waiting‐list controls, or non‐psychological treatments for treating anxiety and depression in children and adolescents with long‐term physical conditions. | i. Real-time: iCBT ii. telemonitoring iii. Hybrid | i. Placebo i. TAU | i. Depression symptoms ii. Anxiety symptoms iii. Treatment acceptability | It could not be determined whether e-health interventions were clearly better than any comparator. For change in depression symptoms versus any control (SMD ‐0.06, 95% CI ‐0.35 to 0.23). For change in anxiety symptoms versus any comparator, (SMD ‐0.07, 95% CI ‐0.29 to 0.14). For treatment acceptability, (SMD 0.46, 95% CI 0.23 to 0.69).For quality of life, (SMD ‐0.83, 95% CI ‐1.53 to ‐0.12). The very low‐quality of the evidence means the effects of e‐health interventions are uncertain at this time, especially in children aged under 10 years. | High | |
| Thomas et al 2014 | 45 | To synthesize literature to evaluate teleglaucoma, its diagnostic accuracy, healthcare system benefits, and cost-effectiveness. | i. Real-time: Teleglaucoma | i. F2F ii. No intervention | i. Cost-effectiveness ii. Diagnostic accuracy | Teleglaucoma is more specific and less sensitive than in-person examination. Pooled estimates of sensitivity was 0.832 [95% CI 0.770, 0.881] and specificity was 0.790 [95% CI 0.668, 0.876]. The relative odds of a positive screen test in glaucoma cases are 18.7 times more likely than a negative screen test in a non-glaucoma cases. Additionally, the mean cost for every case of glaucoma detected was $1098.67 US and of teleglaucoma per patient screened was $922.77 US. As a result teleglaucoma saves costs to patients and costs to the health care system as a whole. | Low | |
| Toma et al 2014 | 34 | To summarise the current evidence surrounding the role of online social networking services (SNS) in diabetes care. | i. Telemonitoring ii. Real time | i. TAU ii. No intervention | i. HbA1c ii. Patient satisfaction iii. Frequency of transmission | Significant reduction in HbA1c favouring the TM intervention group, WMD 0.46% (95% CI [−0.58, −0.34], P < 0.00001). Significant mean difference of −0.45% (95% CI [−0.60, −0.29], P < 0.00001) favouring the intervention group was observed in the change in HbA1c between baseline and follow-up. Online SNS provide a novel, feasible approach to improving glycaemic control, particularly in patients with Type 2 diabetes. | Critically Low | |
| van Beugen, et al 2014 | 23 | To describe and evaluate the effectiveness of guided iCBT interventions for chronic somatic conditions on general psychological outcomes, disease-related physical outcomes, and disease-related impact on daily life outcomes. The role of treatment length was also examined. | i. Real-time and telemonitoring: iCBT (internet-based cognitive behavioural therapy) | i. TAU or F2F ii. information based psychoeducation | i. Depressive symptoms ii. General distress iii. psychological outcomes | Guided iCBT was shown to improve all outcome categories with small effect sizes for psychological outcomes (range 0.17-0.21) and occasionally larger effects for disease-specific physical outcomes (range 0.07 to 1.19) and disease-related impact outcomes (effect size range 0.17-1.11). Interventions with a longer treatment duration (>6 weeks) led to more consistent effects on depression. Guided ICBT appears to be a promising and effective treatment for chronic somatic conditions to improve psychological and physical functioning and disease-related impact. | Critically Low | |
| van Egmond et al 2018 | 23 | To study the effectiveness of physiotherapy with TR on postoperative functional outcomes and quality of life in surgical patients. | TR: i. web-based ii. real-time (telephone) iii. wireless monitored exercise | i. TAU or F2F | i. Functional outcomes ii. Quality of Life (QoL) | 7 meta-analysis, SMD for QoL for was 1.01 (95% CI 0.18,1.84), indicating that QoL increased with TR compared with TAU. The heterogeneity expressed with I2 was high at 97%. Physiotherapy with telerehabilitation has the potential to increase quality of life, is feasible, and is at least equally effective as TAU in surgical populations. | Critically Low | |
| Van Spall et al 2017 | 53 | To compare the effectiveness of transitional care services in decreasing all‐cause death and all‐cause readmissions following hospitalization for heart failure (HF). | i. Telemonitoring: remote monitoring of weight, vital signs or other indices of functional status with or without follow‐up telephone calls | i. TAU or F2F | Clinical outcomes: i. All‐cause mortality and all‐cause readmission rate i. Cost of care | Telemonitoring (9 studies) did not significantly decrease all-cause mortality compared to TAU (RR 0.90, CI 0.68-1.19) nor all cause readmission (IRR 0.82 CI 0.62-1.08). Similarly, telephone support did not decrease all-cause mortality (RR 0.82 CI 0.62-1.08) nor all cause readmission (IRR 0.86 CI 0.64-1.15). | Critically Low | |
| Vigerland 2016 | 25 | To perform a comprehensive, systematic review of the literature in the field of iCBT for children and adolescents and investigate for which childhood psychiatric and somatic conditions ICBT has been tested | i. Telemonitoring ii. Real-time: structured telephone calls to assess patient's clinical condition and provide support | i. TAU | i. Sleep efficiency ii. Depressive symptoms iii. OCD iv. BMI | Twenty-four studies (N = 1882) were included in the meta-analysis and ICBT yielded moderate between-group effect sizes when compared with waitlist, g = 0.62, 95% CI [0.41, 0.84]. The results suggest that CBT for psychiatric and somatic conditions in children and adolescents can be successfully adapted to an internet-delivered format. | Critically Low | |
| Widmer et al 2015 | 51 | To assess the potential benefit of digital health interventions (DHIs) on cardiovascular disease (CVD) outcomes and risk factors compared with non-DHIs. | TM: i. Real-time ii. telemonitoring | i. TAU | i. CVD outcomes including revascularization, stroke, rehospitalization ii. Risk factors including weight loss, BMI, blood pressure, LDL-cholesterol | 9 meta-analysis, digital health interventions significantly reduced CVD outcomes (RR, 0.61; 95% CI, 0.46-0.80; P<.001; I2=22%). Concomitant reductions in weight (−2.77 lb [95% CI, −4.49 to −1.05 lb]; P<.002; I2=97%) and body mass index (−0.17 kg/m2 [95% CI, −0.32 kg/m2 to −0.01 kg/m2]; P=.03; I2=97%) but not blood pressure (−1.18 mm Hg [95% CI, −2.93 mm Hg to 0.57 mm Hg]; P=.19; I2=100%) were found in these DHI trials compared with TAU. | Critically Low | |
| Wootton 2016 | 18 | To synthesize the current literature on remote treatment for OCD using a meta-analytic approach. | i. real-time (video conference, telephone) ii. telemonitoring (Internet delivered cognitive behavioural therapy) | i. Waitlist ii. F2F | i. Symptom reduction | Within-group findings indicate that remote treatment for OCD produces a decrease in symptoms of a large magnitude (g = 1.17; 95% CI: 0.91–1.43). Between-group findings indicate that remote treatment for OCD is more effective than control (g = 1.06; 95% CI: 0.68–1.45) and outcomes are not meaningfully different from F2F treatment (g = − 0.21; 95% CI: − 0.43–0.02). | Critically Low | |
| Zhai et al 2014 | 47 | To evaluate the clinical effectiveness and cost effectiveness of TM approaches on glycemic control in patients with type 2 diabetes mellitus. | i. Real time: telephone ii. Remote monitoring | i. TAU | i. Glycemic control ii. Cost-effectiveness | A small, but statistically significant, decrease in HbA1c following TM intervention was observed compared to conventional treatment (MD = −0.37, 95% CI = −0.49 to −0.25, Z = −6.08, P < 0.001). Optimization of TM approaches could potentially allow for more effective self-management of disease in type 2 diabetes patients, though evidence to-date is unconvincing. | Low | |
| Zhao et al 2015 | 11 | To assess the effectiveness of TM to relieve asthma symptoms | i. Sms ii. Internet based self-management program iii. Telephone counselling | i. TAU ii. F2F | i. asthma symptom score change | 6 meta-analysis, No significant difference in asthma symptom score change between the TM and control groups (pooled g=0.34, 95% CI=−0.05 to 0.74, Z=1.69, p=0.090). TM interventions do not appear to improve asthma function scores, but other benefits may be present. | Critically Low | |
| *TM: Telemedicine, TAU: Treatment as usual or usual care, MD: Mean difference, CI: Confidence interval, TR: Telerehabilitation, SMD: Standard mean difference, WMD: weighted mean difference, HR: Hazard ratio, RR: Relative risk, OR: Odds ratio, HBA1c: Hemoglobin A1c* | | | | | | | | |

# Table 2: Characteristics of Patient Experience Reviews

| **First Author**  **(Year)** | **Included studies (n)** | **Description of review** | **Population/ Setting** | **Outcomes** | **Authors conclusions** | **AMSTAR Review Quality** |
| --- | --- | --- | --- | --- | --- | --- |
| Bashshur (2016) | 86 | To present the evidence for the advantages of TM interventions in primary care | All populations in primary care settings | Feasibility/acceptance (35), intermediate outcomes (36), health-outcomes (7), cost (8) | TM is feasible/acceptable in primary care settings, but varies by population demographics. TM is more acceptable to patients than providers | Critically Low |
| Berrouiguet (2016) | 36 | To review the literature regarding the use of mobile phone text messaging in mental health care. | Patients with mental health conditions | Telemonitoring: text message feasible/acceptable for support (15), self monitoring (15), medication reminders and assessment(5), information (6) | Overall, a positive attitude toward text messages was reported. RCTs reported improved treatment adherence and symptom surveillance. Other positive points included an increase in appointment attendance and in satisfaction with management and health care services. Insight into message content, preventative strategies, and innovative approaches derived from the mental health field may be applicable in other medical specialties. | Critically Low |
| Berry (2016) | 49 | To determine factors that could influence the acceptability of online and mobile phone delivered interventions for severe mental illness (SMI) | Patients with severe mental illness | Acceptability of mobile phone interventions (42) | Telemonitoring via text messaging found to be highly acceptable by patients with SMI | Critically Low |
| Brunton (2015) | 7 | To review and synthesize the findings from qualitative studies that investigated user perspectives and experiences of telehealth in COPD management, in order to identify factors which may impact on uptake. | Patients with COPD | Help-seeking/self-care (5), Risk/safety concerns from health professionals (3), Empowerment (2) | Telehealth technologies have the potential to be beneficial in the management of COPD compared to TAU alone by enabling self-care and empowerment but these benefits may be detrimental through increased risk, dependency and burden. | Critically Low |
| Caffery (2017) | 14 | To examine reported outcomes of health services delivered by telehealth to Indigenous Australians. | Indigenous Australians | Social and emotional wellbeing clinical outcomes, access to health, patient empowerment, health literacy | Telehealth models of care facilitated through partnerships between Aboriginal community-controlled health services and public hospitals may improve both patient outcomes and access to specialist services for Indigenous people. | Critically Low |
| Cox (2017) | 22 | To identify, appraise, and synthesize qualitative research evidence on the experiences of adult cancer survivors participating in telehealth interventions, to characterize the patient experience of telehealth interventions for this group | Adult cancer survivors | Survivor experiences | (1) influence of telehealth on the disrupted lives of cancer survivors (convenience, independence, and burden); (2) personalized care across physical distance (time, space, and the human factor); and (3) remote reassurance—a safety net of health care professional connection (active connection, passive connection, and slipping through the net). Telehealth interventions are convenient and can potentially reduce treatment burden while providing cancer survivors with independence and reassurance. | Critically Low |
| Cruz (2014) | 12 | To provide a comprehensive description of the methodologies used in home telemonitoring interventions for Chronic Obstructive Pulmonary Disease (COPD) and to explore patients’ adherence and satisfaction with the use of telemonitoring systems. | 775 participants, COPD patients | Patient satisfaction, patient training, patient compliance | Patients are overall satisfied with home tele-monitoring systems, however systems need to be adjusted to suit the target population and additional training provided to patients. | Critically Low |
| Gorst (2014) | 37 | To assess levels of uptake of home telehealth by patients with HF and COPD and the factors that determine whether patients do or do not accept and continue to use telehealth. | Adult heart failure or COPD patients | Patients acceptance, abandonment, or perceptions of telehealth. 1) Barriers to telehealth (17) 2) Patient facilitators (27) | Technical problems appeared to be a major issue impacting on the uptake and sustained use of telehealth, with studies reporting little tolerance for poorly working systems, thus it is essential that telehealth equipment is user friendly and functions effectively. Furthermore, users can be unsure of the technology, hence appropriate training and access to support could also support uptake and use. | Low |
| Greenhalgh (2017) | 105 | To inform policy by making sense of a complex literature on heart failure and its remote management. | Adult heart failure patients | Factors that account for low uptake of eHealth: Patient factors, Staff factors, Team/service factors | The limited adoption of telehealth for heart failure has complex clinical, professional and institutional causes, which are unlikely to be elucidated by adding more randomized trials of technology-on versus technology-off to an already-crowded literature. | Critically Low |
| Grist (2017) | 24 | To systematically appraise the available research evidence on the efficacy and acceptability of mobile apps for mental health in children and adolescents younger than 18 | Children and adolescents | 1) Efficacy (5) 2) Feasibility and acceptability (8) | TM interventions delivered through mobile apps is acceptable to children and adolescents with mental health conditions | Critically Low |
| Hamilton (2018) | 9 | To assess the evidence around mHealth interventions for CR and heart failure management for service and patient outcomes, cost effectiveness with a view to how mHealth could be utilized for rural, remote and Indigenous cardiac patients. | Cardiac patients | Acceptability, usage, engagement and adherence | TM is acceptable to cardiac patients with high levels of engagement. Health delivery of CR and heart failure management is feasible with high rates of participant engagement, acceptance, usage and adherence. The efficacy of mHealth in these studies was comparable to traditional center-based CR. mHealth delivery has the potential to improve access to CR and heart failure management for patients unable to attend traditional center-based programs. | Critically Low |
| Liptrott (2018) | 48 | To report adult patients’ perceptions of the acceptability of, and satisfaction with, telephone‐based interventions during or post‐treatment for cancer. | Adult cancer patients | Acceptability, satisfaction, opinions and perceptions | Current evidence relating to the acceptability and satisfaction of support delivered by telephone for cancer patients during or after therapy suggests it is convenient, provides positive personal experiences, enhances accessibility to HCPs and provides a familiar environment in which to facilitate potentially sensitive healthcare discussions. | Low |
| Musiat (2014) | 95 | The aim of this study was to review the evidence of cost-effectiveness, geographical and time flexibility, stigma, therapist time, effects on help-seeking and treatment satisfaction of cCBT interventions for mental health | Mental health patients | Cost-effectiveness, Treatment satisfaction | cCBT interventions are cost-effective and often cheaper than TAU. Limited evidence was found with regard to geographic flexibility, time flexibility, waiting time for treatment, stigma and the effects on help-seeking. Personal support in cCBT was found to take many forms, was not limited only to therapists, and seemed to increase treatment adherence and reduce attrition. Treatment satisfaction with cCBT was found to be high, but more research on attrition due to dissatisfaction is required. | Critically Low |
| Slater (2017) | 12 | To identify, appraise, and synthesize available qualitative evidence on users’ experiences of mHealth technologies for NCD management in young people. We explored the perspectives of both end users (young people) and implementers (health policy makers, clinicians, and researchers). | All qualitative studies on young people (in the age range of 15-24 years) with chronic NCDs (end users) | Experiences, acceptance, perceptions of benefit | Our evidence meta-synthesis revealed both complementary and unique user perspectives on enablers and barriers to designing, developing, and implementing mHealth technologies to support young people’s management of chronic NCDs. mHealth technologies should be considered as a tool to enable self-management, to improve clinical encounters, and to encourage positive health behaviors. Developing mHealth technologies should involve a genuinely collaborative codesign process between end users and implementers, with the capacity to tailor and adapt technologies to meet person-centered needs. This approach will help to ensure meaningful mHealth solutions for young people, while also supporting implementation efforts. | High |
| Trettel (2018) | 204 | The goal was to identify the use and current state of teledermatology across the world with regard to geographical distribution of published studies, treated indications, research questions, and its reliability in diagnosis and therapy compared to classic face‐to‐face consultations | Not specified | Validity, concordance or feasibility (154), Effectiveness (33), Costs/Cost‐effectiveness/Cost–benefits (24), Quality of life (4), Safety (1) | Teledermatology is a reliable consultation tool in the majority of studies. If specified, TM was used in daily dermatological routine for patient management purposes, to consult patients in peripheral locations, or for medical support in nursing homes or home care settings. | Critically Low |

# Table 3: Characteristics of Implementation Reviews

| **First Author (Year)** | **Number of studies** | **Short description of review** | **Population/ Setting** | **Outcomes** | **Findings and authors conclusion** | **AMSTAR Review Quality** |
| --- | --- | --- | --- | --- | --- | --- |
| Alvarado (2017) | 41 | To identify and classify patient barriers to implementing remote health interventions for adult patients with type 2 diabetes in the United States. | Adult patients in the US with type 2 diabetes | i. Low formal education ii. technology illiteracy iii. patient desire for in person contact iv. low perceived value or effectiveness v. health illiteracy vi. technology is cost prohibitive vii. limited internet access in area viii. lack of customization to patient preference and needs | Lack of data accuracy, concerns over scalability and technology illiteracy are the most common barriers to remote health monitoring of Type 2 diabetes patients, leading to declining patient engagement. | Critically Low |
| Block (2016) | 137 | To review of studies using remote physical activity monitoring in neurological diseases, highlighting advances and determining gaps. | Patients with neurological diseases | Physical activity | Emerging evidence supports the feasibility and effectiveness of telemonitoring in neurological care and neurorehabilitation | Critically Low |
| Bradford (2016) | 116 | To review and synthesise the available literature regarding telehealth services in rural and remote locations of Australia, and to identify the factors associated with their sustained success. | People living in rural and remote Australia | Factors influencing success and sustainability of tele-health services: vision, ownership, adaptability, economics, efficiency and equipment | Telehealth has the potential to address many of the key challenges to providing health in Australia, with its substantial land area and widely dispersed population. | Low |
| Bruce (2018) | 16 | To present evidence on the use of teledermoscopy to improve the accuracy of skin lesion identification in adult populations. | Adults with skin lesions | i. Diagnostic accuracy i. Diagnostic reliability i. Feasibility | There is limited evidence on the effectiveness of teledermascopy in the accurate diagnosis of skin lesions mainly due to variation in instruments used to capture skin lesion images | Critically Low |
| Cruz (2014) | 12 | To provide a comprehensive description of the methodologies used in home telemonitoring interventions for Chronic Obstructive Pulmonary Disease (COPD) and to explore patients’ adherence and satisfaction with the use of telemonitoring systems. | 775 participants, COPD patients | Patient satisfaction (9), patient training (9), patient compliance (5) | Patients are overall satisfied with home tele-monitoring systems; however systems need to be adjusted to suit the target population and additional training provided to patients. | Critically Low |
| Gehring (2017) | 29 | To identify implementation foci in research studies and government/organizational documents for eMental healthcare technologies for paediatric mental healthcare. | Children (0-18) for included studies, all populations for government documents | Acceptability (14), Adoption (5), appropriateness (10), cost (20), feasibility (2), fidelity (2), penetration, and sustainability (8 govt. documents) | Studies have largely focused on acceptability and appropriateness, while government/organizational documents state goals and recommendations regarding costs, feasibility, and sustainability of eMental healthcare technologies. These differences suggest that the research evidence available for paediatric eMental healthcare technologies does not reflect the focus of governments and organizations. Partnerships between researchers, healthcare planners, and policymakers may help to align implementation research with policy development, decision-making, and funding foci | Low |
| Greenwood (2014) | 15 | To summarize research on telehealth remote patient monitoring interventions that incorporate key elements of structured self-monitoring of blood glucose (SMBG) identified as essential for improving A1C. | Diabetic (type 2) patients using insulin | Satisfaction, Adherence, haemoglobin A1C | Telehealth RPM interventions that incorporate more key elements of structured SMBG appear to have the greatest impact on A1C. It is critical to incorporate purposeful SMBG profiles that allow the individual to change behaviour or the PCP to modify treatment. Engaging persons with diabetes in self-management requires education, an understanding of SMBG profiles and goals, and the opportunity for interactive feedback as they engage in behaviour change. | Critically Low |
| Guise (2014) | 22 | To identify patient safety risks associated with telecare use in homecare services and to investigate whether and how these patient safety risks have been addressed in telecare training. | Adults receiving care at home | Tele homecare systems: evaluation, acceptability, user experiences, implementation. Change in the nature of clinical work (15); Lack of patient and/or staff knowledge and understanding (13); Technology issues (9); Changes to staff workload (8); Accessibility issues (3); Lack of guidelines (3); Patient dependency (3); Patient anxiety (2); Poor system integration (2); Poor patient compliance (2); and nature of homecare environment (1). | There is a need to better identify and describe patient safety risks related to telecare services to improve understanding of how to avoid and minimize potential harm to patients. This process can be aided by reframing known telecare implementation challenges and user experiences of telecare with the help of a human factors systems approach to patient safety. | Critically Low |
| Irving (2018) | 39 | To explore the quantitative and qualitative framework associated with teledentistry in an effort to uncover the interaction of multiple influences on its delivery and sustainability. |  | Main themes identified: (1) using information and communication technology (ICT), (2) regulatory and system improvements, (3) accuracy of teledentistry, (4) effectiveness, including increasing access to clinical services, efficiencies and acceptability, and (5) building and increasing clinical capacity of the dental workforce. | Teledentistry provides a viable option for remote screening, diagnosis, consultation, treatment planning and mentoring in the field of dentistry. Rapidly developing information and communication technologies have increasingly shown improving cost effectiveness, accuracy and efficient remote assistance for clinicians. There is high acceptability for teledentistry amongst clinicians and patients alike. Remuneration of advising clinicians is critical to sustainability. | Critically Low |
| Ito (2018) | 42 | To systematically review the Japanese and English language literature relating to the clinical use of TM in Japan. | Not specified | screening, diagnosis (16), | Japan is actively seeking opportunities to use TM in addressing the healthcare needs of the population. Key focus areas are 1) access to healthcare for rural and remote communities and 2) home TM. Also, the clinically related studies reflect the Japanese cultural focus on disease prevention and surveillance. | Critically Low |
| Kampmeijer (2016) | 45 | A systematic review of the evidence on the scope of the use of e-health and m-health tools in health promotion and primary prevention among older adults (age 50+). | Older adults (50+) |  | The successful use of e-health/m-health tools in health promotion programs for older adults greatly depends on the older adults’ motivation and support that older adults receive when using e-health and m-health tools. | Critically Low |
| Kapadia (2015) | 58 | To identify the key issues that affect the adoption of ICT in the aged care sector | Older adults | Reliability, usability, cost, health conditions, perceived need for technology, social isolation, confidentiality and security | ICT empowers health professionals to improve operational efficiency, reduce medical errors, and increase capacity for managing limited resources effectively. The availability of ICT enables older people stay independent longer in their home. A key to success for aged people adopting technology is usability and cost. Therefore, developing low-cost and user-friendly technology for older people is the first step towards the successful implementation of ICT | Critically Low |
| Macdonald (2018) | 48 | To explore the enablers and barriers faced by adults with diabetes using two-way information communication technologies to support diabetes self-management. | Adults with diabetes | 1) Technology usage 2) Poor interface and technology design 3) Environment, access and supportive infrastructure | People with diabetes face a number of potentially modifiable barriers in using technology to support their diabetes management. In order to address these barriers, end users should be consulted in the design process and consideration given to theories of technology adoption to inform design and implementation. | Critically Low |
| McDougall (2017) | 20 | To identify and summarize the published and grey literature on the use of TM for the diagnosis and management of inflammatory and/or autoimmune rheumatic disease. | Autoimmune and rheumatology patients | 1) Cost-effective (6) 2) Diagnosis by telerhematology effective (7) | Most data relate to the management of inflammatory arthritis during follow-up. Studies to date are at high risk of bias, use predominately VTC TRh, and include physicians as the patient presenter. TM reporting methods varied widely, and rigorous cost analyses are lacking. Most studies viewed TRh favourably, with 1 notably stating a potential for harm. | Critically Low |
| Meurk (2016) | 30 | To review e-mental health service use for depressive and anxiety disorders to inform policy development and identify policy-relevant gaps in the evidence base |  | 1) facilitating uptake (17) 2) Treatment preferences (6) 3) Governing mechanisms (23) | Successfully establishing e-mental health care within the health system will depend on the skilful coordination of activities within clinical, community, research and development, and policy-making realms. | Low |
| Radhakrishnan (2016) | 16 | To identify the barriers and facilitators for sustainability of tele‐homecare programs implemented by home health nursing agencies for chronic disease management. | Not specified | Barriers and facilitators for sustainability of telehomecare, main themes: 1) perceptions of effectiveness 2) tailoring to patients 3) nurse-patient communication and collaboration 4) interpersonal communication and collaboration 5) organisation of process and culture 6) quality of tele homecare technology | The findings of this systematic review provide implications for sustained usage of tele‐homecare programs by home health nursing agencies and can help such programs realize their potential for chronic disease management. | Critically Low |
| Wickramasinghe (2016) | 14 | To identify enablers and barriers associated with the delivery of telehealth services for diabetes care amongst Indigenous people. | Indigenous diabetic patients | Enablers: 1) Telehealth trained local staff 2) Preintervention community engagement 3) Audio-visual material included Barriers: 1) Poor quality images 2) Delayed follow-up 3) Scheduling issues, lack of staff training | In the right circumstances, the delivery of tele diabetes services is promising, especially in circumstances where specialist services are not available or difficult to access. | Low |

# Table 4: Country of origin of primary studies included in reviews

| **Country*** | **Studies (n)** | **Studies (%)** |
| --- | --- | --- |
| **United States** | 534 | 36.7% |
| **Australia** | 251 | 17.2% |
| **United Kingdom** | 135 | 9.3% |
| **Japan** | 75 | 5.2% |
| **Canada** | 67 | 4.6% |
| **Netherlands** | 59 | 4.1% |
| **Spain** | 43 | 3.0% |
| **Italy** | 27 | 1.9% |
| **Korea** | 26 | 1.8% |
| **Sweden** | 23 | 1.6% |
| **Germany** | 21 | 1.4% |
| **Austria** | 20 | 1.4% |
| **Finland** | 15 | 1.0% |
| **Norway** | 15 | 1.0% |
| **Denmark** | 13 | 0.9% |
| **Brazil** | 9 | 0.6% |
| **New Zealand** | 9 | 0.6% |
| **Taiwan** | 9 | 0.6% |
| **China** | 8 | 0.5% |
| **Belgium** | 7 | 0.5% |
| **Portugal** | 7 | 0.5% |
| **India** | 7 | 0.5% |
| **Ireland** | 6 | 0.4% |
| **Serbia** | 6 | 0.4% |
| **South Korea** | 6 | 0.4% |
| **France** | 5 | 0.3% |
| **Poland** | 5 | 0.3% |
| **Switzerland** | 5 | 0.3% |
| **Malaysia** | 5 | 0.3% |
| **Hong Kong SAR** | 4 | 0.3% |
| **Iran** | 3 | 0.2% |
| **Israel** | 3 | 0.2% |
| **Mexico** | 3 | 0.2% |
| **Thailand** | 3 | 0.2% |
| **Kenya** | 2 | 0.1% |
| **Tanzania** | 2 | 0.1% |
| **Czech Republic** | 2 | 0.1% |
| **Greece** | 2 | 0.1% |
| **Turkey** | 2 | 0.1% |
| **Singapore** | 2 | 0.1% |
| **Mali** | 1 | 0.1% |
| **South Africa** | 1 | 0.1% |
| **Bulgaria** | 1 | 0.1% |
| **Croatia** | 1 | 0.1% |
| **Lithuania** | 1 | 0.1% |
| **Belize** | 1 | 0.1% |
| **Colombia** | 1 | 0.1% |
| **Guatemala** | 1 | 0.1% |
| **Honduras** | 1 | 0.1% |
| **Indonesia** | 1 | 0.1% |
| **Total** | 1456 | 100.0% |

*An additional 44 studies originated in Europe, 29 in North America and 15 in Asia.
