# Supplementary material for: Telemedicine in the OECD: An umbrella review of clinical and cost-effectiveness, patient experience and implementation
Source: PLoS One. 2020 Aug 13;15(8):e0237585. doi: 10.1371/journal.pone.0237585 (PMC7425977; doi:10.1371/journal.pone.0237585)
Supplement: S3 File — (DOCX) [file pone.0237585.s003.docx]

| **Methodological Quality of Included Systematic Reviews** | | | | | | | | | | | | | | | | | | |  |
| --- | --- | --- | --- | --- | --- | --- | --- | --- | --- | --- | --- | --- | --- | --- | --- | --- | --- | --- | --- |
| **Review First Author (Year)** | | **Q1** | **Q2** | **Q3** | **Q4** | **Q5** | **Q6** | **Q7** | **Q8** | **Q9** | **Q10** | **Q11** | **Q12** | **Q13** | **Q14** | **Q15** | **Q16** | **Overall Quality** |  |
| Adamse (2018) | | Y | N | Y | Y | Y | Y | N | Y | Y | Y | Y | Y | Y | Y | Y | Y | Critically Low |  |
| Agostini (2015) | | Y | N | Y | Y | Y | Y | Y | Y | Y | Y | Y | Y | Y | Y | Y | Y | Low |  |
| Akiyama (2016) | | Y | N | Y | P | N | Y | N | Y | Y | Y | N/A | N/A | Y | Y | N/A | Y | Critically Low |  |
| Alvarado (2017) | | Y | N | Y | Y | Y | Y | N | Y | N | N | N/A | N/A | Y | Y | N/A | Y | Critically Low |  |
| Bashshur (2016) | | Y | N | Y | P | U | U | N | Y | N | N | N/A | N/A | N | Y | N/A | Y | Critically Low |  |
| Berrouiguet (2016) | | Y | N | Y | P | U | U | N | Y | N | N | N/A | N/A | N | Y | N/A | Y | Critically Low |  |
| Berry (2016) | | Y | N | Y | Y | Y | Y | N | y | N | N | N/A | N/A | Y | Y | N/A | Y | Critically Low |  |
| Block (2016) | | Y | N | Y | P | Y | Y | N | Y | Y | Y | N/A | N/A | N/A | Y | N/A | Y | Critically Low |  |
| Bradford (2016) | | Y | Y | Y | Y | Y | Y | N | Y | Y | N | N/A | N/A | N/A | N | N/A | Y | Low |  |
| Bruce (2018) | | Y | N | Y | P | U | N | N | Y | Y | N | N/A | N/A | N/A | Y | N/A | Y | Critically Low |  |
| Brunton (2015) | | Y | N | Y | P | Y | Y | N | Y | Y | N | N/A | N/A | N/A | Y | N/A | Y | Critically Low |  |
| Caffery (2017) | | Y | Y | Y | Y | Y | Y | N | Y | N | N | N/A | N/A | N | N | N/A | Y | Critically Low |  |
| Cottrell (2017) | | Y | Y | Y | Y | Y | Y | N | Y | Y | N | Y | Y | Y | Y | N | Y | Critically Low |  |
| Cox (2017) | | Y | N | Y | P | Y | Y | N | Y | Y | N | N/A | N/A | N | Y | N/A | Y | Critically Low |  |
| Cruz (2014) | | Y | N | Y | P | U | Y | N | Y | Y | N | Y | Y | Y | Y | Y | Y | Critically Low |  |
| Cruz (2014) | | Y | N | Y | P | N | N | N | Y | Y | N | N/A | N/A | Y | Y | N/A | Y | Critically Low |  |
| Dario (2017) | | Y | Y | Y | Y | Y | Y | N | Y | Y | N | Y | Y | Y | Y | Y | Y | Low |  |
| de la Torre-Díez (2015) | | Y | N | Y | P | N | N | N | Y | N | N | N/A | N/A | Y | Y | N/A | Y | Critically Low |  |
| Deady (2017) | | Y | N | Y | Y | Y | Y | N | Y | Y | N | Y | Y | Y | Y | Y | Y | Critically Low |  |
| Direito (2017) | | Y | P | Y | Y | U | U | N | Y | Y | N | Y | Y | Y | Y | Y | Y | Low |  |
| Estai (2018) | | Y | N | Y | P | Y | Y | N | Y | Y | N | N/A | N/A | Y | Y | N/A | Y | Critically Low |  |
| Feltner (2014) | | Y | Y | Y | Y | Y | Y | N | Y | Y | Y | Y | Y | Y | Y | Y | Y | Low |  |
| Flodgren (2015) | | Y | Y | Y | Y | Y | Y | Y | Y | Y | Y | Y | Y | Y | Y | Y | Y | High |  |
| Fuertes-Guiró (2017) | | Y | N | Y | P | Y | Y | N | Y | Y | N | Y | Y | Y | Y | Y | Y | Critically Low |  |
| Gehring (2017) | | Y | P | Y | Y | Y | Y | N | Y | Y | N | N/A | N/A | Y | Y | N/A | Y | Low |  |
| Gorst (2014) | | Y | N | Y | P | Y | N | Y | Y | Y | N | N/A | N/A | Y | Y | N/A | Y | Low |  |
| Greenhalgh (2017) | | Y | N | Y | Y | Y | Y | N | Y | N | N | N/A | N/A | Y | Y | N/A | Y | Critically Low |  |
| Greenwood (2014) | | Y | N | Y | P | Y | Y | N | Y | N | N | N/A | N/A | Y | Y | N/A | Y | Critically Low |  |
| Grist (2017) | | Y | N | Y | Y | U | U | N | Y | N | N | N/A | N/A | N | N | N/A | Y | Critically Low |  |
| Grustam (2014) | | Y | N | Y | Y | Y | Y | N | Y | Y | N | N/A | N/A | Y | Y | N/A | Y | Critically Low |  |
| Guise (2014) | | Y | N | Y | P | Y | Y | N | Y | Y | N | N/A | N/A | Y | Y | N/A | Y | Critically Low |  |
| Hakala (2017) | | Y | Y | Y | Y | Y | Y | Y | Y | Y | N | Y | Y | Y | Y | N | Y | Low |  |
| Hameed (2014) | | Y | N | Y | P | Y | Y | Y | Y | N | N | N/A | N/A | Y | Y | N/A | Y | Critically Low |  |
| Hamilton (2018) | | Y | N | Y | P | Y | Y | N | Y | Y | N | N/A | N/A | N | Y | N/A | Y | Critically Low |  |
| Huang (2014) | | Y | Y | Y | Y | Y | Y | Y | Y | Y | Y | Y | Y | Y | Y | N/A | Y | High |  |
| Huang (2015) | | Y | N | Y | Y | Y | Y | N | Y | Y | N | Y | Y | Y | Y | N | Y | Critically Low |  |
| Huang (2015) | | Y | N | Y | P | Y | Y | N | Y | Y | N | Y | Y | Y | Y | Y | Y | Critically Low |  |
| Hui (2017) | | Y | Y | Y | Y | Y | Y | N | Y | Y | N | Y | Y | Y | Y | N | Y | Critically Low |  |
| Hutchesson (2015) | | Y | Y | Y | P | Y | Y | N | Y | Y | N | Y | Y | Y | Y | Y | Y | Low |  |
| Iribarren (2017) | | Y | Y | Y | Y | Y | Y | Y | Y | Y | N | N/A | N/A | Y | Y | N/A | Y | High |  |
| Irving (2018) | | Y | N | Y | P | Y | Y | N | Y | Y | N | N/A | N/A | Y | Y | N/A | Y | Critically Low |  |
| Ito (2017) | | Y | N | Y | P | Y | Y | N | Y | N | N | N/A | N/A | Y | Y | N/A | Y | Critically Low |  |
| Jackson (2016) | | Y | N | Y | P | Y | Y | N | Y | Y | N | N/A | N/A | Y | Y | N/A | Y | Critically Low |  |
| Jeon (2015) | | Y | N | Y | P | Y | Y | N | Y | N | N | Y | Y | N | N | N | Y | Critically Low |  |
| Joiner (2017) | | Y | N | Y | P | N | Y | N | Y | Y | N | Y | Y | Y | Y | Y | Y | Critically Low |  |
| Kampmeijer (2016) | | Y | N | Y | P | U | U | N | Y | N | N | N/A | N/A | Y | Y | N/A | Y | Critically Low |  |
| Kapadia (2015) | | Y | N | Y | P | Y | Y | N | Y | N | N | N/A | N/A | Y | Y | N/A | Y | Critically Low |  |
| Kelly (2016) | | Y | Y | Y | Y | Y | Y | N | Y | Y | N | Y | Y | Y | Y | Y | Y | Low |  |
| Kepplinger (2016) | | Y | Y | Y | Y | Y | Y | N | Y | Y | N | Y | Y | Y | Y | Y | Y | Low |  |
| Klersy (2016) | | Y | Y | Y | Y | Y | Y | Y | Y | Y | N | Y | Y | Y | Y | Y | Y | High |  |
| Kotb (2015) | | Y | N | Y | P | Y | Y | N | Y | Y | N | Y | Y | Y | Y | N | Y | Critically Low |  |
| Lee (2016) | | Y | N | Y | P | Y | Y | N | Y | N | N | Y | Y | N | N | N | Y | Critically Low |  |
| Lee (2018) | | Y | Y | Y | P | Y | Y | N | Y | Y | N | Y | Y | Y | Y | N | Y | Critically Low |  |
| Liddy (2016) | | Y | Y | Y | Y | U | U | N | Y | Y | N | N/A | N/A | Y | Y | N/A | Y | Low |  |
| Linde (2015) | | Y | Y | Y | Y | Y | Y | N | Y | Y | N | Y | Y | Y | Y | Y | Y | Low |  |
| Liptrott (2018) | | Y | P | Y | P | N | Y | N | Y | Y | N | N/A | N/A | Y | Y | N/A | Y | Low |  |
| Liu (2017) | | Y | N | Y | P | U | U | N | Y | Y | N | Y | Y | Y | Y | N | Y | Critically Low |  |
| López-Villegas (2016) | | Y | N | Y | Y | Y | Y | Y | Y | Y | N | N/A | N/A | Y | Y | N/A | Y | Low |  |
| Lundell (2015) | | Y | Y | Y | P | Y | Y | N | Y | Y | Y | Y | Y | Y | Y | Y | Y | Low |  |
| Macdonald (2018) | | Y | N | Y | P | Y | Y | N | Y | Y | N | N/A | N/A | Y | Y | N/A | Y | Critically Low |  |
| Marx (2018) | | Y | Y | Y | P | Y | Y | N | Y | Y | Y | Y | Y | Y | Y | Y | Y | Low |  |
| McDougall (2017) | | Y | Y | Y | Y | Y | N | N | Y | N | N | N/A | N/A | Y | Y | N/A | Y | Critically Low |  |
| McLean (2016) | | Y | Y | Y | Y | Y | Y | N | Y | Y | N | Y | Y | Y | Y | Y | Y | Low |  |
| Merriel (2014) | | Y | N | Y | P | Y | Y | N | Y | Y | N | Y | Y | Y | Y | Y | Y | Critically Low |  |
| Meurk (2016) | | Y | P | Y | Y | Y | Y | Y | Y | N | N | N/A | N/A | Y | Y | N/A | Y | Low |  |
| Michaud (2018) | | Y | Y | Y | P | Y | Y | N | Y | Y | Y | N/A | N/A | Y | Y | N/A | Y | Low |  |
| Ming (2016) | | Y | P | Y | P | Y | Y | N | Y | Y | N | Y | Y | Y | Y | N/A | Y | Low |  |
| Musiat (2014) | | Y | N | Y | P | Y | Y | N | Y | N | N | N/A | N/A | Y | Y | N/A | Y | Critically Low |  |
| Nair (2018) | | Y | Y | Y | P | Y | Y | N | Y | Y | N | Y | Y | Y | Y | N/A | Y | Low |  |
| Oosterveen (2017) | | Y | Y | Y | P | Y | Y | N | Y | Y | N | Y | Y | Y | Y | Y | Y | Low |  |
| Radhakrishnan (2016) | | Y | N | Y | P | Y | Y | N | Y | Y | N | N/A | N/A | Y | Y | N/A | Y | Critically Low |  |
| Raman (2017) | | Y | Y | Y | Y | Y | Y | Y | Y | Y | Y | Y | Y | Y | Y | Y | Y | High |  |
| Rasekaba (2015) | | Y | P | Y | P | Y | Y | Y | Y | Y | Y | Y | Y | Y | Y | N/A | Y | High |  |
| Rawstorn (2016) | | Y | P | Y | P | Y | Y | N | Y | Y | N | Y | Y | Y | Y | N | Y | Low |  |
| Sanyal (2018) | | Y | N | Y | Y | Y | Y | N | Y | Y | Y | N/A | N/A | Y | Y | N/A | Y | Critically Low |  |
| Seiler (2017) | | Y | Y | Y | P | Y | Y | N | Y | Y | N | Y | Y | Y | Y | Y | Y | Low |  |
| Seyffert (2016) | | Y | N | Y | P | Y | Y | N | Y | Y | N | Y | Y | Y | Y | Y | Y | Critically Low |  |
| Sherifali (2017) | | Y | N | Y | P | Y | Y | N | Y | Y | N | Y | Y | Y | Y | N | Y | Critically Low |  |
| Slater (2017) | | Y | Y | Y | Y | Y | Y | Y | Y | Y | N | N/A | N/A | Y | Y | N/A | Y | High |  |
| Snoswell (2016) | | Y | Y | Y | Y | Y | Y | N | Y | Y | N | N/A | N/A | Y | Y | N/A | Y | Low |  |
| Speyer (2018) | | Y | N | Y | P | Y | Y | N | Y | Y | N | Y | Y | Y | Y | Y | Y | Critically Low |  |
| Stratton (2017) | | Y | N | Y | P | Y | Y | N | Y | Y | N | Y | Y | Y | Y | Y | Y | Critically Low |  |
| Su (2016) | | Y | N | Y | P | Y | Y | N | Y | N | N | Y | Y | Y | Y | Y | Y | Critically Low |  |
| Tchero (2017) | | Y | N | Y | P | Y | Y | N | Y | Y | Y | Y | Y | Y | Y | Y | Y | Critically Low |  |
| Thabrew (2018) | | Y | Y | Y | Y | Y | Y | Y | Y | Y | Y | Y | Y | Y | Y | Y | Y | High |  |
| Thomas (2014) | | Y | N | Y | Y | Y | N | Y | Y | Y | Y | Y | Y | Y | Y | Y | Y | Low |  |
| Toma (2014) | | Y | N | Y | P | Y | Y | N | Y | Y | N | Y | Y | Y | Y | Y | Y | Critically Low |  |
| Trettel (2018) | | Y | N | Y | P | U | U | N | Y | N | N | N/A | N/A | N | Y | N/A | Y | Critically Low |  |
| Udsen (2014) | | Y | N | Y | Y | U | U | N | Y | Y | N | N/A | N/A | Y | Y | N/A | Y | Critically Low |  |
| van Beugen (2014) | | Y | N | Y | P | Y | Y | N | Y | Y | N | Y | Y | Y | Y | N | Y | Critically Low |  |
| van Egmond (2018) | | Y | N | Y | P | Y | Y | N | Y | Y | N | Y | Y | Y | Y | N/A | Y | Critically Low |  |
| Van Spall (2017) | | Y | N | Y | P | Y | Y | N | Y | Y | N | Y | Y | Y | Y | Y | Y | Critically Low |  |
| Vigerland (2016) | | Y | N | Y | P | Y | Y | N | Y | Y | N | Y | Y | Y | Y | Y | Y | Critically Low |  |
| Wickramasinghe (2016) | | Y | Y | Y | P | Y | Y | N | Y | Y | N | N/A | N/A | Y | Y | N/A | Y | Low |  |
| Widmer (2015) | | Y | N | Y | P | Y | Y | N | Y | Y | N | Y | Y | Y | Y | Y | Y | Critically Low |  |
| Wootton (2016) | | Y | N | Y | P | N | N | N | Y | Y | N | Y | Y | Y | Y | Y | Y | Critically Low |  |
| Zhai (2014) | | Y | N | Y | P | Y | Y | Y | P | Y | N | Y | Y | Y | Y | Y | Y | Low |  |
| Zhao (2015) | | Y | N | Y | P | Y | Y | N | Y | Y | N | Y | Y | Y | Y | Y | Y | Critically Low |  |
| **Assessment Questions** | | | | | | | | | | | | | | | | | | |  |
| Critical domains (*grey highlight*) | Q2: Protocol registration Q4: Adequacy of the literature search Q7: Justification for excluding studies  Q9: Risk of bias from studies included in review Q11: Appropriateness of meta-analytical methods  Q13: Consideration of risk of bias in interpretation of results Q15: Assessment of presence and likely impact of publication bias. | | | | | | | | | | | | | | | | | |  |
|  |  |  |  |  |  |  |  |  |  |  |  |  |  |  |  |  |  |  |  |
|  |  |  |  |  |  |  |  |  |  |  |  |  |  |  |  |  |  |  |  |
| Non-critical domains (*no highlight*) | Q1: Inclusion of PICO elements in review question Q3: Explain selection of study design Q5: Duplicate study selection Q6: Duplicate data extraction Q8: Description of studies Q10: Report sources of funding for primary studies Q12: Impact of risk of bias assessment on evidence Q14: Explanation for heterogeneity Q16: Report potential conflicts of interest and funding sources by review authors. | | | | | | | | | | | | | | | | | |  |
|  |  |  |  |  |  |  |  |  |  |  |  |  |  |  |  |  |  |  |  |
|  |  |  |  |  |  |  |  |  |  |  |  |  |  |  |  |  |  |  |  |
|  |  |  |  |  |  |  |  |  |  |  |  |  |  |  |  |  |  |  |  |
| **Grading criteria** | | | | | | | | | | | | | | | | | | |  |
| Y (Yes): Criterion met; "P" (Partial yes): Criterion partly met; "N" (No): Criterion not met; "N/A" (Not applicable), U (Unclear) | | | | | | | | | | | | | | | | | | |  |
| High Quality | No or one non-critical weakness | | | | | | | | | | | | | | | | | |  |
| Moderate Quality | More than one non-critical weakness | | | | | | | | | | | | | | | | | |  |
| Low Quality | One critical flaw with or without non-critical weakness | | | | | | | | | | | | | | | | | |  |
| Critically Low Quality | More than one critical flaw with or without non-critical weakness | | | | | | | | | | | | | | | | | |  |
